# Evidence of Omics, Immune Infiltration, and Pharmacogenomic for SENP1 in the Pan-Cancer Cohort

**DOI:** 10.3389/fphar.2021.700454

**Published:** 2021-07-01

**Authors:** Somayye Taghvaei, Farzaneh Sabouni, Zarrin Minuchehr

**Affiliations:** ^1^ Department of Medical Biotechnology, National Institute of Genetic Engineering and Biotechnology, Tehran, Iran; ^2^ Department of Systems Biotechnology, National Institute of Genetic Engineering and Biotechnology, Tehran, Iran

**Keywords:** SENP1, immune infiltration, cell cycle, tumorigenesis, FDA- approved drugs, TCGA

## Abstract

Sentrin specific-protease 1 (SENP1) is a protein involved in deSUMOylation that is almost overexpressed in cancer. SENP1 has a determinative role in the activation of transcription programs in the innate immune responses and the development B of and C lymphocytes. We found, SENP1 possibly plays a critical role in immune infiltration and acts as an expression marker in PAAD, ESCA, and THYM. CD4^+^ T cells, CD8^+^ T cells, and macrophages were more key-related immune cells, indicating that SENP1 might be introduced as a potential target for cancer immunotherapy. We further showed that dysregulation of SENP1 is powerfully associated with decreased patient survival and clinical stage. Total SENP1 protein also increases in cancer. SENP1 is also controlled by transcription factors (TFs) CREB1, KDM5A, REST, and YY1 that regulates apoptosis, cell cycle, cell proliferation, invasion, tumorigenesis, and metastasis. These TFs were in a positive correlation with SENP1. MiR-138–5p, miR-129-1-3p, and miR-129-2-3p also inhibit tumorigenesis through targeting of SENP1. The *SENP1* expression level positively correlated with the expression levels of UBN1, SP3, SAP130, NUP98, NUP153 in 32 tumor types. SENP1 and correlated and binding genes: SAP130, NUP98, and NUP153 activated cell cycle. Consistent with this finding, drug analysis was indicated SENP1 is sensitive to cell cycle, apoptosis, and RTK signaling regulators*.* In the end, SENP1 and its expression-correlated and functional binding genes were enriched in cell cycle, apoptosis, cellular response to DNA damage stimulus. We found that the cell cycle is the main way for tumorigenesis by SENP1. SENP1 attenuates the effect of inhibitory drugs on the cell cycle. We also introduced effective FDA-Approved drugs that can inhibit SENP1. Therefore in the treatments in which these drugs are used, SENP1 inhibition is a suitable approach. This study supplies a wide analysis of the SENP1 across The Cancer Genome Atlas (CGA) cancer types. These results suggest the potential roles of SENP1 as a biomarker for cancer. Since these drugs and the drugs that cause to resistance are applied to cancer treatment, then these two class drugs can use to inhibition of SENP1.

## Introduction

There are seven sentrin specific-protease (SENP) isoforms that operate with SUMO 1–3 (SENP one to three and 5–8). The SENPs are complicated in the deSUMOylation from their substrate proteins and in the maturation of SUMO (Hang and Dasso, 2002). SENP1 (NM_001267594.2 for mRNA or NP_001254523.1 for protein) contains the C-terminal domain that shows catalytic activity and the N-terminal domain that regulates cell localization and substrate specificity.

In mammalian cells, SENPs are differently located. SENP1 (HGNC:17,927) is located at the PML bodies and can commonly act on all the SUMOs one to three precursors (Nayak and Müller, 2014). SENP1 upregulation is a comparatively preliminary event in prostate carcinogenesis. SENP1 enhances the transcriptional activity of AR, eases c-Jun dependent transcription, and induces expression of the cell cycle regulator (Cyclin D1) (Bawa-Khalfe and Yeh, 2010). The overall dynamics of SUMOylation/deSUMOylation may be changed by cell growth, cell cycle conditions, and disease state and SENP proteins might have an important role in cancer growth and be an appropriate target for cancer treatment and therapy. *SENP1* deletion has been prevented cell growth by upregulation of CDK inhibitors, such as p21 and p16 *in vitro* and *in vivo* growth of colon cancer cells (Xu et al., 2011). Prostate cancer cell growth could be induced, because HIF1α activation and stabilization by SENP1 results in promoted Cyclin D1 and VEGF levels, angiogenesis, and cell growth (Cheng et al., 2006). SENP1 organizes matrix metalloproteinase 2 (*MMP2*) and *MMP9* expressions. This introduces SENP1 in the progression of prostate cancer and suggests SENP1 as a prognostic marker and a therapeutic target for prostate cancer metastasis patients (Wang et al., 2013a). SENP1 can also cause lung, breast, and bladder cancers (Brems-Eskildsen et al., 2010; Wang et al., 2013b; Wang et al., 2016). *SENP1* was upregulated in pancreatic ductal adenocarcinoma (PAAD) tissues compared with adjacent normal tissues. The positive dependency of SENP1 with lymph node metastasis and TNM Classification of Malignant Tumors (TNM) stage was exhibited by clinical data. Silencing of *SENP1* leads to *MMP-9* downregulation, which is fundamental for PAAD cell growth and migration (Ma et al., 2014)**.** SENP1 can be utilized as a molecular target in the discovery of anti-tumor drugs vs. human hepatocellular carcinoma (HCC) metastasis. Zhang *et al.* indicated *SENP1* knockdown leads to inhibition of HGF-induced proliferation and migration of HCC at the same time (Zhang et al., 2016). SENP1 is reported to be involved in hepatocarcinogenesis through the regulation of HIF-1α deSUMOylation in hypoxia conditions. Novel inhibitor development that particularly targets SENP1 may offer a new therapeutic approach to block development, metastasis, and recurrence of HCC (Cui et al., 2017). Increased expression of *SENP1* has been also reported in thyroid adenomas (Jacques et al., 2005). These studies suggest SENP1 has a main role in carcinogenesis.

TCGA data collection referred to as the “Pan-Cancer” dataset, presents the scientific community with data on DNA alterations, gene expression, survival status, methylation status, immune infiltration, pharmacogenomics, and protein abundances to detect tumorigenesis effects in different cancer types (Mercatelli et al., 2019). The aim of the present study was the demonstration of SENP1 importance and identification of molecular mechanisms and functions of SENP1 and its interacted proteins in carcinogenesis. There is yet no sufficient Pan-Cancer evidence on the relation between SENP1 and different tumor types based on clinical data. Thus, we conducted a Pan-Cancer study on SENP1 protein. We suggested SENP1 through the effect on cell cycle can result in cancer. The findings of this study indicate the important role of SENP1 in carcinogenesis and supply a potential relationship and a mechanism between SENP1 and tumor-immune interactions. Besides, we represented drugs for SENP1 inhibition. We reported additional references for future experimental studies on SENP1 in cancer.

## Material and Methods

### Genetic Alterations

In order to study genetic alterations of the SENP1 gene in the Pan-Cancer cohort, including ACC (Adrenocortical carcinoma), BLCA (Bladder Urothelial Carcinoma), BRCA (Breast invasive carcinoma), CESC (Cervical squamous cell carcinoma and endocervical adenocarcinoma), CHOL (Cholangiocarcinoma), COAD (Colon adenocarcinoma), DLBC (Lymphoid Neoplasm Diffuse Large B-cell Lymphoma), ESCA (Esophageal carcinoma), GBM (Glioblastoma multiforme), HNSC (Head and Neck squamous cell carcinoma), KICH (Kidney Chromophobe), KIRC (Kidney renal clear cell carcinoma), KIRP (Kidney renal papillary cell carcinoma), LAML (Acute Myeloid Leukemia), LGG (Brain Lower Grade Glioma), LIHC (Liver hepatocellular carcinoma), LUAD (Lung adenocarcinoma), LUSC (Lung squamous cell carcinoma), MESO (Mesothelioma), OV (Ovarian serous cystadenocarcinoma), PAAD (Pancreatic adenocarcinoma), PCPG (Pheochromocytoma and Paraganglioma), PRAD (Prostate adenocarcinoma), READ (Rectum adenocarcinoma), SARC (Sarcoma), SKCM (Skin Cutaneous Melanoma), STAD (Stomach adenocarcinoma), TGCT (Testicular Germ Cell Tumor), THCA (Thyroid carcinoma), THYM (Thymoma), UCEC (Uterine Corpus Endometrial Carcinoma), UCS (Uterine Carcinosarcoma), and UVM (Uveal Melanoma), we used cBioPortal portal (https://www.cbioportal.org/) (Cerami et al., 2012; Gao et al., 2013) through the “TCGA Pan-Cancer Atlas Studies” in the “Quick select” section and entered “SENP1” as query. The results of the genetic alterations, including mutation type, and CNA (Copy number alteration) in the TCGA tumor samples were identified in the “Cancer Types Summary” module. The “Comparison” module was also applied to gain the survival data on the overall, disease-specific, progression-free, and disease-free survival differences for the TCGA cancers with or without SENP1 genetic alteration. Log-rank *p*-value < 0.05 was applied.

### Gene Expression Analysis

The “Expression analysis-Box Plots” module of the GEPIA2 (Gene Expression Profiling Interactive Analysis, version 2) webserver (http://gepia2.cancer-pku.cn/#analysis) (Tang et al., 2019) was used to determine the expression profile of SENP1 between the tumor tissues (9,664 samples), the normal of tumor tissues (711 samples) from The Cancer Genome Atlas (TCGA), and the normal tissues from the Genotype-Tissue Expression (GTEx) database (4,829 samples) as box plot, under “Match TCGA normal and GTEx data”, log_2_FC (fold change) cutoff = 1, and *p*-value cutoff = 0.01 among 33 cancer types.

We also applied the UALCAN portal (http://ualcan.path.uab.edu/analysis-prot.html), to carry out *SENP1* expression analysis from the CPTAC (Clinical proteomic tumor analysis consortium) dataset (Chen et al., 2019). The expression level of the total protein between primary tumor and normal tissues was explored through entering “SENP1”. The available CPTAC datasets were included breast cancer, colon cancer, ovarian cancer, clear cell RCC (renal cell carcinoma), UCEC (Uterine corpus endometrial carcinoma), LUAD (Lung adenocarcinoma), and Pediatric Brain Cancer.

We also obtained stage plots of the *SENP1* expression in various pathological stages (stage I- IV) of all the TCGA tumors using the “Pathological Stage Plot” module of GEPIA2. The log_2_ [TPM (Transcripts per million) +1] expression data and AdjP-value<0.05 were used to draw the stage plots.

### Regulatory Networks of Sentrin Specific-Protease 1 (Transcription Factors, miRNAs, and Methylation)

#### Transcription Factors and miRNAs

To study epigenetic alterations of SENP1, TFs with binding ability to the *SENP1* promoter were anticipated using Harmonizome (https://maayanlab.cloud/Harmonizome) (Rouillard et al., 2016), including CHEA Transcription Factor Targets, ENCODE Transcription Factor Targets, JASPAR Predicted Transcription Factor Targets, and TRANSFAC Curated Transcription Factor Targets databases. Then for TFs with at least two repetitions, their correlation with SENP1 were calculated using ENCORI (http://starbase.sysu.edu.cn/panCancer.php) (Li et al., 2014) database (*p*-value < 0.05, R = 1 to −1).

MiRNAs were considered to post-transcriptionally regulate the expression of more than 60% of the human genome through targeting their 3′ untranslated regions (3′UTR) and affect cell proliferation, apoptosis, and invasion in cancer (Yu et al., 2013). MiRNAs that regulate SENP1 were predicted using the miRWalk (http://mirwalk.umm.uni-heidelberg.de/) (Sticht et al., 2018), including miRDB, miRTarBase, and TargetScan databases. Then for miRNAs with at least two repetitions, their correlation with SENP1 was calculated using ENCORI (http://starbase.sysu.edu.cn/panCancer.php) (Li et al., 2014) database (*p*-value < 0.05, R = 1 to −1).

#### Methylation

We investigated methylation of SENP1 in the TCGA cancers using the DNMIVD (http://119.3.41.228/dnmivd/index/) (Ding et al., 2020). The correlation between methylation and expression of *SENP1* in the TCGA cancers and the correlation of differential survival with methylation state of SENP1 were obtained in the TCGA cancers through the DNMIVD database.

### Immune Infiltration Analysis

We also investigated the correlation between *SENP1* expression levels and immune cell infiltration levels via TIMER2 (http://timer.cistrome.org/) (Li et al., 2020) across different cancer types. The correlation between *SENP1* expression and immune infiltration in 21 immune cell types in the 32 TCGA tumors were visualized using the “Immune-Gene” module of the TIMER2. The TIMER, CIBERSORT, CIBERSORT-ABS, QUANTISEQ, XCELL, MCPCOUNTER, TIDE, and EPIC algorithms were used for immune infiltration estimations. The data were visualized as a heatmap. *p*-values < 0.05 and −1<R < 1 were obtained viz the Spearman’s rank correlation test and were considered statistically significant.

### Survival Prognosis Analysis

The “Survival Map” module of the GEPIA2 (Tang et al., 2019) was applied to assess the OS (Overall survival)and DFS (Disease-free survival) survival map of SENP1 in the 33 TCGA cancers. We classified tumors into two groups: the low-expression and high-expression groups. We also utilized the “Survival Analysis” module of the GEPIA2 for OS and DFS plots using the log-rank test in the hypothesis test. *p*-value < 0.05 which was considered statistically significant.

### Sentrin Specific-Protease 1-Related Gene Enrichment Analysis

We drew a protein-protein interaction network for SENP1 through STRING (Doncheva et al., 2018) App in Cytoscape version 3.8.1 (Kohl et al., 2011; Saito et al., 2012) using the query (“SENP1”) and organism (“*Homo sapiens*”). In the following, we set the main parameters: minimum required interaction score [“medium confidence (0.4)”], and max additional interactors 100 for obtaining SENP1-binding proteins.

We drew an interactive functional association network for SENP1 using GeneMANIA (Montojo et al., 2010) App in Cytoscape version 3.8.1 (Kohl et al., 2011; Saito et al., 2012) with max resultant genes 100 and max resultant attributes 100. We also applied the “Similar Gene Detection” module of the GEPIA2 to get the top 100 SENP1-correlated targeting genes based on expression in all the TCGA tumor samples. Using 100 correlated genes, we drew an expression-correlated network for SENP1 by STRING (Doncheva et al., 2018) App in Cytoscape version 3.8.1 (Kohl et al., 2011; Saito et al., 2012) that contains members with correlated expression to SENP1.

Then an intersection analysis was conducted using between STRING network and the GeneMANIA network with the correlated network by Venn (http://bioinformatics.psb.ugent.be/webtools/Venn/) and five common genes (UBN1, SP3, SAP130, NUP98, and NUP153) were obtained between two functional networks with expression network. Four genes were common between the correlated network and the GeneMANIA network, two genes were common between the correlated network and STRING network, and one gene was common between three networks. Then correlation of five common genes with SENP1 in the Pan-Cancer cohort were calculated using ENCORI database (http://starbase.sysu.edu.cn/panCancer.php) (Li et al., 2014) (*p*-value < 0.05, −1<R < 1).

Enrichr (Chen et al., 2013) is a tool for gene enrichment analysis that estimates the importance of overlap between an input list of genes and the gene sets in existing gene libraries. In the end, we uploaded the STRING network, GeneMANIA network, and correlated network to Enrichr for GO ontology and KEGG pathway. We considered *p*-value < 0.01 statistically significant.

### Drug and Pathway Analysis for Sentrin Specific-Protease 1 in the Pan-Cancer Cohort, Virtual Screening, and Molecular Docking

#### Drug and Pathway Analysis for Sentrin Specific-Protease 1 in the Pan-Cancer Cohort

We also downloaded data for SENP1-associated drugs from PharmacoDB (https://www.pharmacodb.pmgenomics.ca/) (Smirnov et al., 2018; Smirnov et al., 2017), *p*-value < 0.01 and standardized coefficient −1 to 1. We obtained targets and target pathways of drugs with statistical significance through the Genomics of Drug Sensitivity in Cancer (GDSC) database https://www.cancerrxgene.org/) (Yang et al., 2012). Pathway activity pie chart and heatmap were obtained from GSCALite (http://bioinfo.life.hust.edu.cn/web/GSCALite/) (Liu et al., 2018) in the Pan-Cancer cohort.

#### Virtual Screening of Potential Compounds Targeting Sentrin Specific-Protease 1

A designing inhibitor for SENP1 can distinguish some clues for the cancer treatment and assist the functional study of SENP1 at the molecular level for experimental biologists. We selected FDA-Approved drugs of the ZINC15 database (http://zinc.docking.org/ ) (Sterling and Irwin, 2015) as the library. All the drug-like compounds were gotten from the ZINC15 database in SDF format. We used PyRx 0.8 (Dallakyan and Olson, 2015) for structure-based virtual screening (SBVS). We received 2IYC from RCSB Protein Data Bank (https://www.rcsb.org/) (Deshpande et al., 2005) as PDB form. 2IYC converted into PDBQT format. The SDF file was imported in the open babel of PyRx and energy minimization of all the ligands was done. Then all compounds were converted into AutoDock PDBQT format. The search space encompassed with dimensions in Å: center (x, y, z) = (27.9890, −9.5475, −6.1959), dimensions (x, y, z) = (250,000, 250,000, 250,000). Thus, the ligands were docked with SENP1 protein using AutoDock Vina (Trott and Olson, 2010) in PyRx 0.8 (Dallakyan and Olson, 2015).

#### Molecular Docking by AutoDock Tools

After the virtual screening, we selected twenty top results for molecular docking. Then, before initiating the docking operation, the protein and ligand structure were prepared. Molecular docking of SENP1 was performed with twenty top FDA-Approved compounds from virtual screening results using AutoDock4 software (Morris et al., 2009). Binding position includes the coordinates x center = 33.658 -y center = −16.605 -z center = −0.55 and active site amino acids include TRP465, LEU466, HIS529, GLY531, VAL532, HIS533, TRP534, MET552, GLN596 and CYT603. Polar hydrogen atoms were incorporated by the hydrogen module in AutoDock4 (ADT) for SENP1. Non-polar hydrogens were merged. Gasteiger charges were added. Docking protocol was performed in a grid box consisting of 60 × 60 × 60 (x, y, z) points at the center and with a grid resolution of 0.375 Å to cover SENP1 binding site. Docking was performed with a genetic algorithm. 25 × 10^5^ energy evaluations with a maximum of 27,000 generations number were performed. The population size was fixed at 150 in each run, mutation rate at 0.02, and cross-over the rate at 0.80. For the ligands, the torsions were defined using the “Ligand torsions” menu option of AutoDock Tools. Other parameters were set to default amounts (Taghvaei et al., 2021). In the end, compounds with the lowest binding energy in SBVS and molecular docking by AutoDock4 were visualized using Discovery Studio (Studio, 2008).

## Results

### Genetic Alterations

**FIGURE 1:**
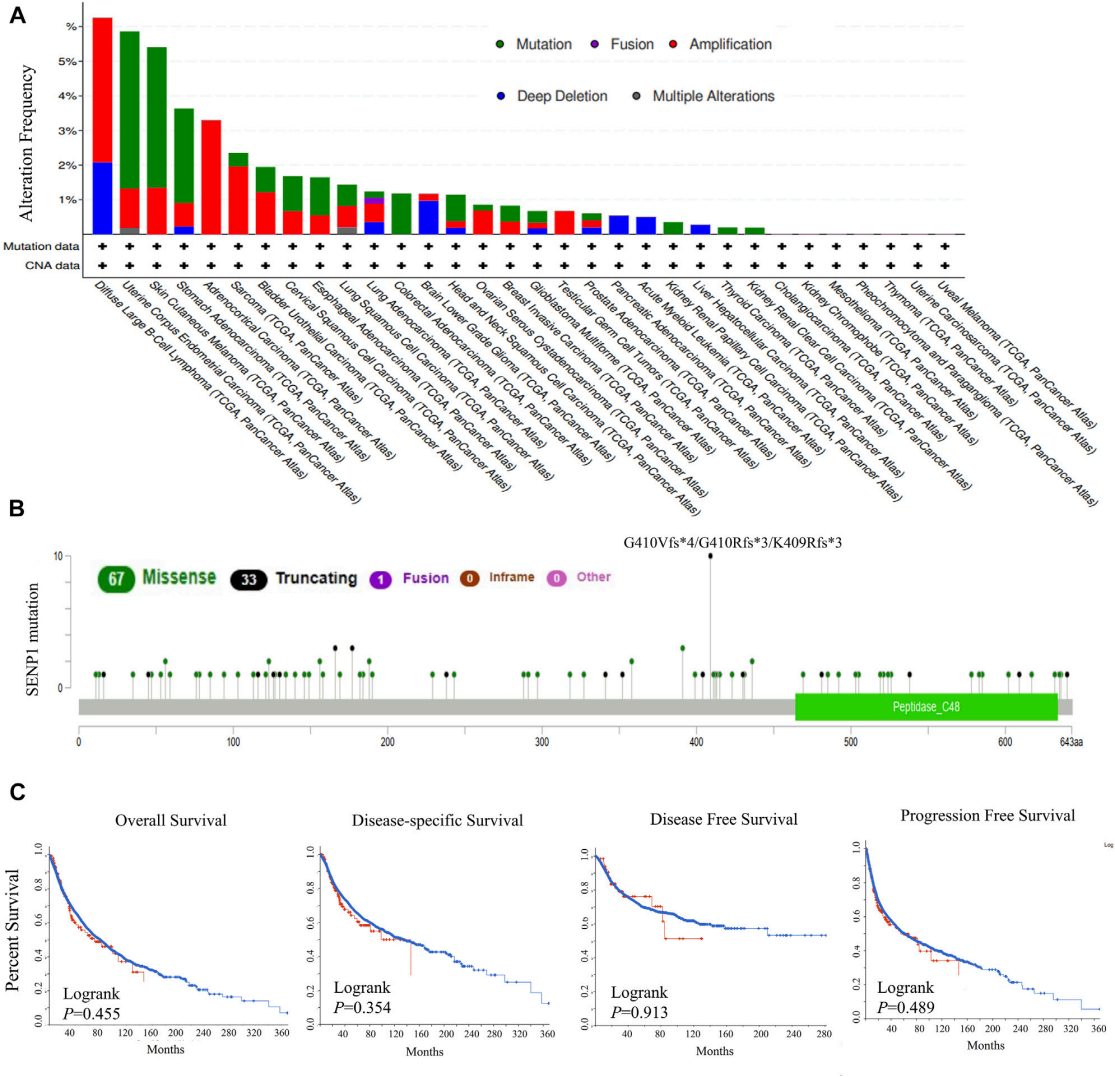
Genetic alterations in SENP1, **(A)** Mutation type and copy number alterations, **(B)** Mutation types in SENP1 with the most frequency of missense mutations, and **(C)** OS, DSS, DFS, and PFS in cancers with genetic alterations.

At first, we explored genetic alterations of SENP1 in the 33 tumors, including 10,969 tumor samples. The highest genetic alteration rates were for amplification type in DLBC, ACC, and SARC with frequencies of 4.2, 3.3, and 2%, respectively (Figure 1A). Another genetic alteration was mutation with frequencies of 4.5, 4, and 2.7% in UCEC, SKCM, and STAD, respectively (Figure 1A). Among the mutations, missense mutations had the highest frequency (Figure 1B). Deep deletion was also observed with 2% frequency in DLBC. Fusion and multiple alterations were with lower frequency than 1%, (Figure 1A). We observed mutations in the peptidase domain which contains the active site of SENP1 and is essential for SENP1 function (Figure 1B). Generally, across tumor types, the genetic alterations rate was 1.5% and the mutation rate was only 0.8%.

Also, no genetic alteration of *SENP1* had effect on the survival prognosis of the TCGA tumors. Because we found disease-free (*p* = 0.913), disease-specific (*p* = 0.354), overall (*p* = 0.355), and progression-free (*p* = 0.489) survivals were not statistically significant, (Figure 1C). We concluded that the percentage of genetic alterations in the study of SENP1 was negligible.

### Differential Expression Analysis Sentrin Specific-Protease 1 Between Tumor and Normal Samples

**FIGURE 2:**
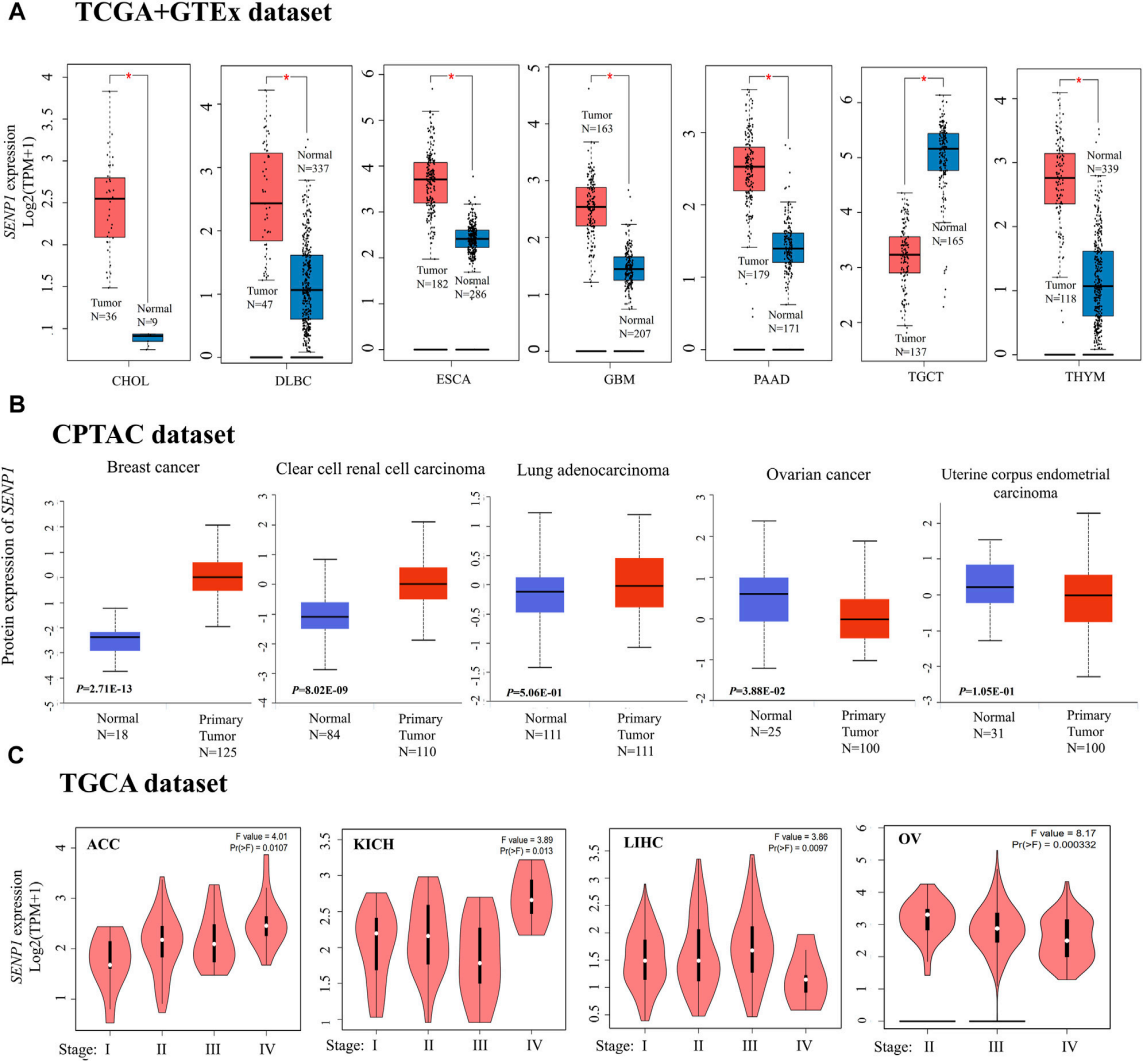
*SENP1* expression, **(A)** Box plots of *SENP1* expression in tumor tissues compared with normal tissues, **(B)** SENP1 total protein in tumor tissues compared with normal tissues, and **(C)** *SENP1* expression in the different stages of cancers ACC, KICH, LIHC, and OV.

We carried out differential expression analysis of the 33 TCGA cancers using GEPIA2 (Figure 2A). The results were demonstrated significant differential expression between cancer tissues and para-cancerous tissues. Data of the TCGA shown higher expression of *SENP1* in tumor tissues of CHOL, DLBC, ESCA, GBM, PAAD, and THYM than normal tissues, but lower expression of *SENP1* in tumor tissues than normal tissues in TGCT (Figure 2A, *p-*value < 0.01). Other cancers were displayed in Supplementary Figure 1.

The CPTAC dataset also indicated higher expression of *SENP1* total protein in the primary tissues of breast cancer, clear cell RCC (renal cell carcinoma), and LUAD (Lung adenocarcinoma) (Figure 2B, *p* < 0. 01) than normal tissues. While we observed higher expression of *SENP1* total protein in the normal tissues of ovarian cancer and UCEC (Uterine corpus endometrial carcinoma) compared with primary cancer tissues.

The results of the Pathological Stage Plot of GEPIA2 also displayed the correlation between SENP1 expression and the pathological stages of cancers consisting of only ACC (*p* < 0.0107), KICH (*p* < 0.013), LIHC (*p* < 0.0097), and OV (*p* < 0.000332) (Figure 2C, *p* < 0.05). Other cancers were displayed in Supplementary Figure 2.

### Transcription Factors and miRNAs Controls Sentrin Specific-Protease 1

#### Transcription Factors and miRNAs

**FIGURE 3:**
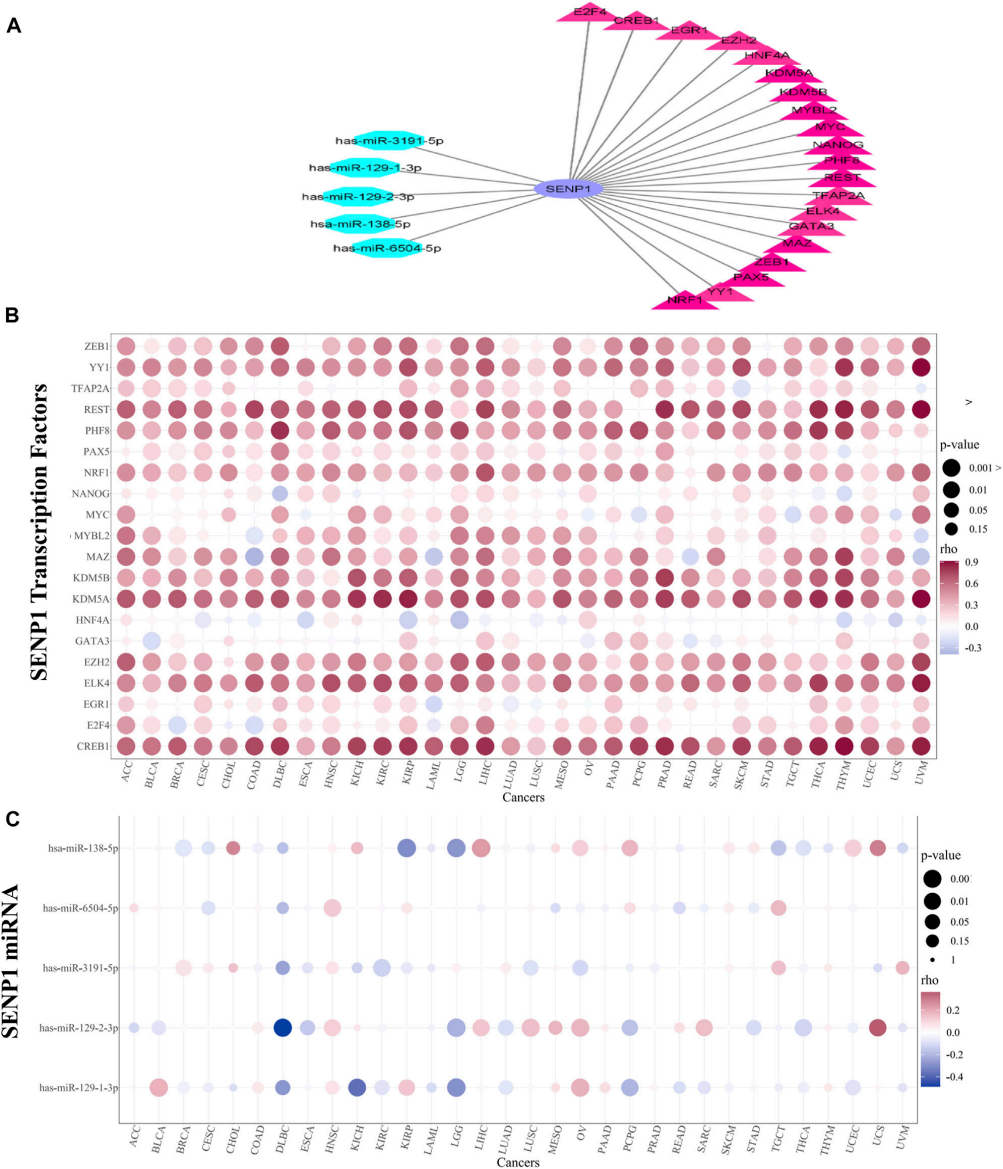
TF and miRNA regulatory networks. **(A)** TF and miRNA network, **(B)** Bubble plot of TFs alterations in 32 cancers, and **(C)** Bubble plot of miRNAs alterations in 32 cancers.

During mitosis, transcription factors could keep the capacity to bind to their targets and nucleosomal arrays (Festuccia et al., 2019). Due to the significance of SENP1 in cancer, we explored transcription factors and miRNAs that regulate SENP1. We obtained 149 TFs regulating SENP1 in four databases consisting of CHEA, NCODE, JASPAR, and TRANSFAC. Twenty TFs were common in two or more than two databases and were selected. These TFs were displayed as a bubble plot based on correlation in 32 tumors (Figure 3B). CREB1 in THYM (R = 0.903), KDM5A in UVM (R = 0.914), REST in UVM (R = 0.909), and YY1 in UVM (R = 0.908) had the most positive correlation with SENP1. CREB1 regulates apoptosis, cell cycle, cell proliferation, invasion, carcinogenesis, and metastasis and decreases drug sensitivity *in vitro* (Ye et al., 2017). CREB1 targets the Bcl2 family, Cyclins, and Egr-1 (early growth response 1 (Yan et al., 2018). KDM5A inhibition causes anticancer activity through impaired cell cycle and senescence via regulation of p16 and p27. KDM5A leads to inhibition of p^RB^ function and P53 signaling in the cell cycle. KDM5A can be a target to treat cancer because it triggers tumorigenesis (Shokri et al., 2018). YY1 is an important regulator in tumorigenesis that its expression was disturbed in many tumors. YY1 can cause activation of oncogenes and suppression of tumor suppressors (Arribas et al., 2015). Overexpression and activation of *YY1* are related to uncontrollable cell proliferation, resistance to apoptosis stimulators, carcinogenesis, and metastasis (Gordon et al., 2006). REST causes apoptosis, decreasing cell proliferation and Bcl2 expression, and increasing drug sensitivity (Lv et al., 2010). Then SENP1 interacts with these TFs and can through them contribute in the carcinogenesis.

miRNAs can regulate gene expression at the post-transcriptional level and affect cell proliferation, apoptosis, and invasion in cancer (Li et al., 2018). MiRNAs that can control *SENP1* were also gotten from miRWalk (consisting of miRDB, miRTarBase, and TargetScan). From eight miRNAs, five miRNAs (has-miR-129-1-3p, has-miR-2-3p, has-miR-138–5p, has-miR-6504–5p, and has-miR-3191–5p) were common in two or more of two databases that have been shown as bubble plot in Figure 3C. The TF and miRNA network were drawn in Cytoscape and were demonstrated in Figure 3A. We concluded that miR-138–5p in Kidney Renal Papillary Cell Carcinoma (KIRP) (R = -0.369), miR-129-1-3p in Kidney Chromophobe (KICH) (R = -0.304), and miR-129-2-3p in Lymphoid Neoplasm Diffuse Large B-cell Lymphoma (DLBC) (R = -487) have the most inhibitory effects on SENP1 expression (Figure 3C).

The previous studies reported the miRNA-138–5p can inhibit the progression of lung adenocarcinoma, and (head and neck squamous cell carcinoma (HNSCC), and prostate, colorectal, breast, ovarian, and gastric cancers, (Zhang et al., 2020). The miRNA-129-1-3p can prevent tumor progression in glioblastoma, and colorectal, and gastric cancers. The miRNA-129-2-3p can also inhibit tumor progression in hepatocellular carcinoma (HCC), and glioblastoma, and colorectal, breast, gastric cancers (Yu et al., 2013).

The inhibition of SENP1 by miRNA-129-1-3p and miRNA-129-2-3p were reported in any study. Since these miRNAs inhibit tumorigenesis and affect SENP1 expression, then we can use their overexpression for inhibition of SENP1.

#### Methylation

Methylation of SENP1 in 32 cancers were also considered in the DNMIVD. SENP1 regulation in cancer was not affected by methylation. Only in BRCA (*p*-value = 0.0154,259, R = −0.182,279) and COAD (*p*-value = 0.00935,263, R = 0.103,032) were observed significant correlation that were no significant change in the methylation level, *p-*value < 0.05, Spearman_r between one and −1. We concluded promoter DNA methylation cannot control the expression of SENP1 in cancer.

### Sentrin Specific-Protease 1 Expression is Correlated With Immune Infiltration in Pancreatic Adenocarcinoma, Esophageal Carcinoma, Lymphoid Neoplasm Diffuse Large B-cell Lymphoma, and Thymoma

One of the cancer hallmarks is the immune reaction. Solid tumors are generally infiltrated with immune cells consisting of B and T lymphocytes, macrophages, eosinophils, neutrophils, mast cells, NK-T cells, natural killer (NK) cells, dendritic cells (DCs), etc. Infiltration of cells is responsible for chronic inflammation. Increased data demonstrated that local inflammation powerfully induces cancer development (Pagès et al., 2010).

**FIGURE 4:**
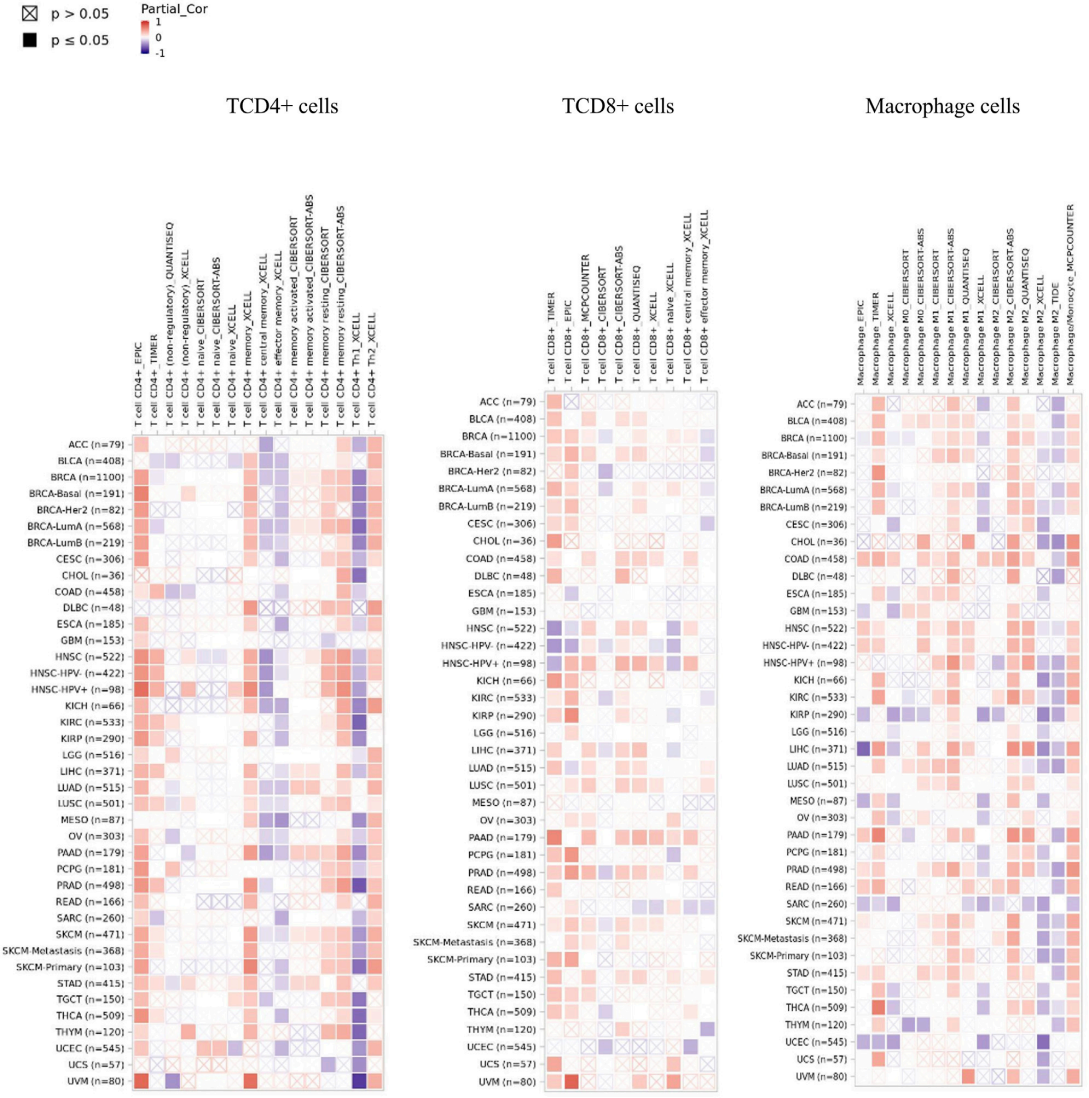
Heatmap of immune cells with positive significant correlation with SENP1 in the Pan-Cancer cohort.

*SENP1* expression was demonstrated significant positive correlations with B cells in 31 cancers, CD4^+^ T cells in 31 cancers, neutrophils in 31 cancers, macrophage cells in 30 cancers, DC in 29 cancers, CD8^+^ T cells in 29 cancers, Tregs in 27 cancers, NK cells in 26 cancers, mast cells in 24 cancers, and monocytes in 23 cancers. We observed a general pattern from a positive correlation between *SENP1* expression and immune cell infiltration. *SENP1* expression level was shown significant positive correlations with abundances of infiltrating immune cells, including B cells, CD4^+^ T cells, CD8^+^ T cells, DC cells, NK cells, Tregs, monocytes, and macrophages. Then SENP1 can play an important role in cancer. Also, we obtained the top ten of immune cells with significantly positive correlations that among they, immune cells with correlation≥4 were consisting of CD4^+^ T cells, CD8^+^ T cells, monocytes, and macrophages in PAAD, DC cells in ESCA, NK cells in DLBC, and monocytes in THYM according to all the applied algorithms. Among these cells, CD4^+^ T cells, CD8^+^ T cells, and macrophages were more key-related immune cells that have been displayed in Figure 4, indicating that SENP1 can increase the tumor-infiltrating immune cell abundance in cancer. Other cells were displayed in Supplementary Figure 2. According to our results, *SENP1* expression had significant expression changes and higher immune infiltration in PAAD, ESCA, and THYM.

### Survival Analysis Data

**FIGURE 5:**
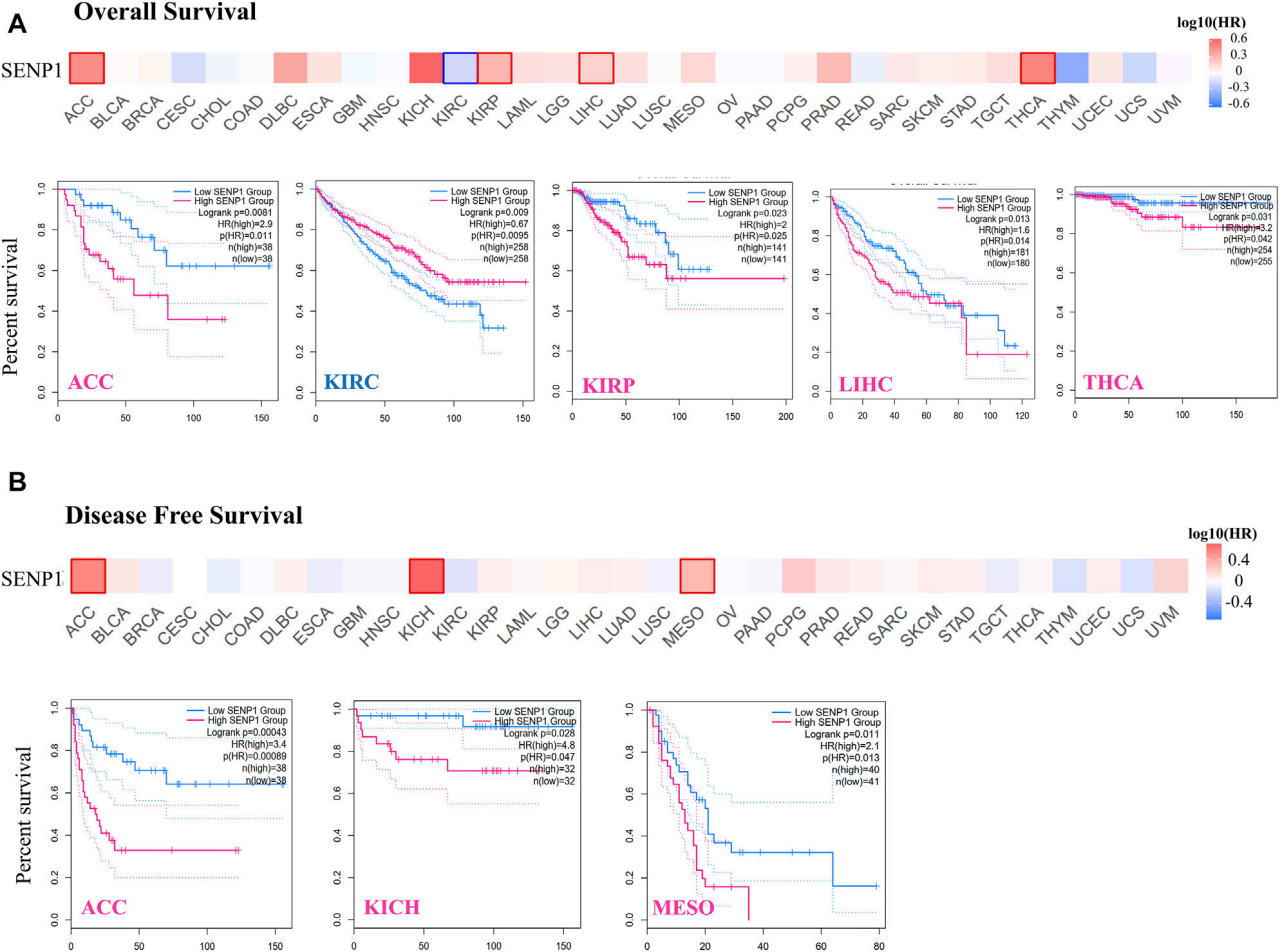
Survival of patients with the altered *SENP1* expression in various tumors, **(A)** Overall survival of SENP1 in ACC, KIRC, KIRP, LIHC, and THCA, and **(B)** Disease-Free Survival of SENP1 in ACC, KICH, and MESO.

GEPIA2 also was applied to obtain survival of the TCGA tumors. In the survival analysis, the cancer cases were divided into high-expression and low-expression groups based on the expression levels of SENP1 and the correlation of SENP1 expression with the prognosis of patients of various tumors, principally through the TCGA datasets were explored. High expression of SENP1 was associated with a significantly worse prognosis of OS in ACC (*p* = 0.0081, HR = 2.9), KIRP (*p* = 0.023, HR = 2), LIHC (*p* = 0.013, HR = 1.6), and THCA (*p* = 0.031, HR = 3.2) of the TCGA (Figure 5A). DFS data analysis has represented a correlation between high expression of SENP1 and significantly worse prognosis in the ACC (*p* = 0.00043, HR = 3.4), KICH (*p* = 0.028, HR = 4.8), LIHC (*p* = 0.026, HR = 1.2), and MESO (Figure 5B, *p* = 0.011, HR = 2.1). Moreover, low SENP1 expression was associated with poor OS prognosis of KIRC (Figure 5B, *p* = 0.0095, HR = 0.67). These results propose the potential role of SENP1 as a marker for cancer survival. Other tumors with *p* > 0.05 were shown in the Supplementary Figure 4.

### Enrichment of Sentrin Specific-Protease 1-Binding Proteins Networks

**FIGURE 6:**
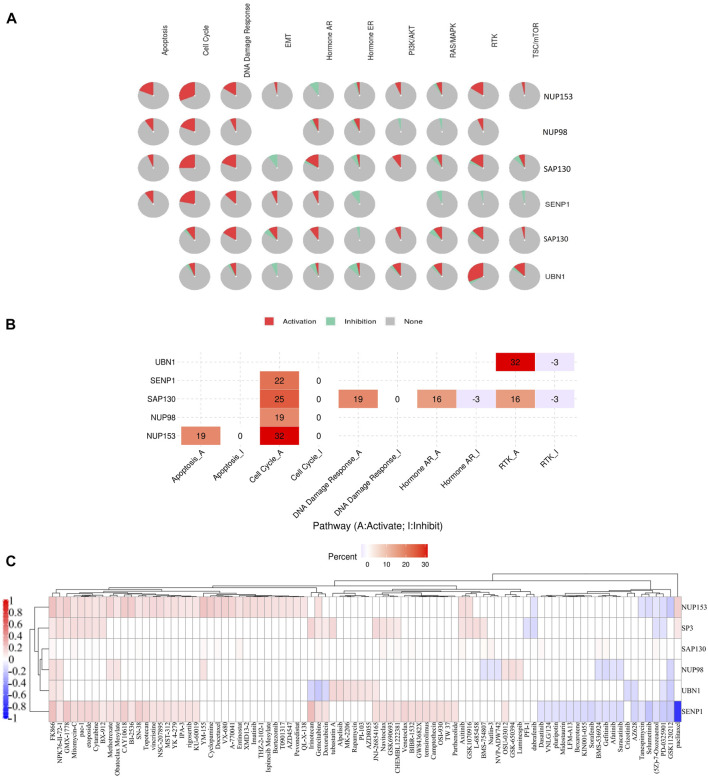
SENP1 network. **(A)** SENP1-correlated network, **(B)** Venn diagram of SENP1-binding genes of STRING, and GeneMANIA with SENP1-correlated genes, and **(C)** Correlation heatmap of common five genes with SENP1 in 32 cancers, **(D)** Go ontology (BP), and KEGG pathway of SENP1-correlated network.

Due to the importance of SENP1 in cancer. We drew the expression-correlated network for SENP1 (Figure 6A). We also drew functional networks of STRING and GeneMANIA (Supplementary Figure 5 and Supplementary Figure 6). The five genes: UBN1, SP3, SAP130, NUP98, and NUP153 were common between functional networks and expression-correlated networks (Figure 6B, Venn diagram). Correlation heatmap of these genes with SENP1 has been shown in the Pan-Cancer cohort in Figure 6C. We observed a correlation with all tumor samples in 32 TCGA cancers.

KEGG pathway enrichment results shown “Cell Cycle” and “Apoptosis” might be complicated in the effect of SENP1 on tumorigenesis. The GO enrichment analysis data also represented that these three networks involved in the cellular response to DNA damage stimulus, regulation of the apoptotic process, and regulation of cell cycle (Figure 6D and Supplementary Figure 5 and Supplementary Figure 6). Based on these results, we concluded the potential involvement of SENP1 in tumor progression.

### Drug and Pathway Activity Analysis Shown a Role of Sentrin Specific-Protease 1 in the Pan-Cancer Cohort

#### Drug and Pathway Activity Analysis

In the next step to more study and the survey of SENP1 function during cancer, we explored the correlation between expression of five common genes and SENP1 with activation or inhibition of signaling pathways.

**FIGURE 7:**
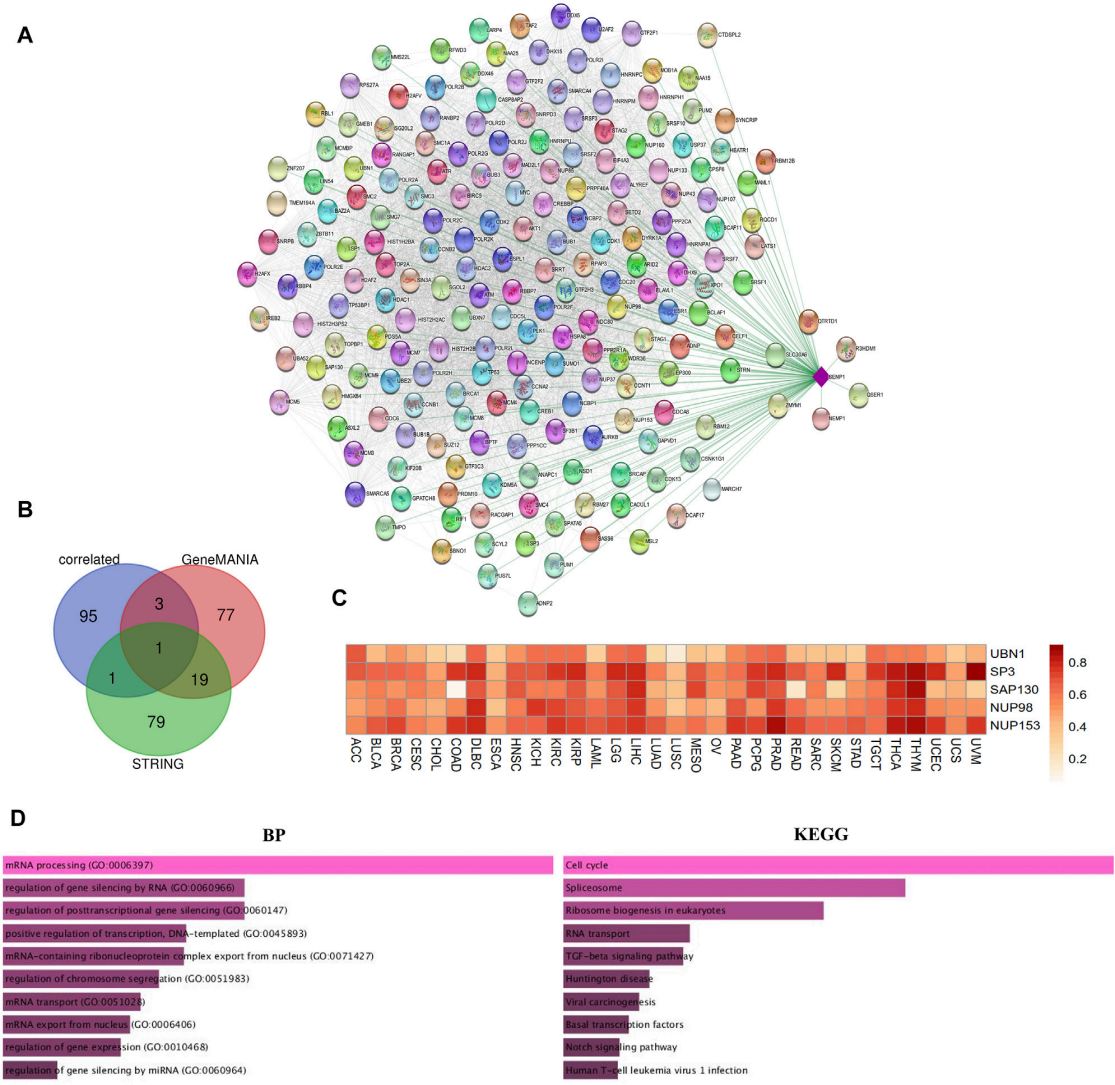
The role of SENP1 in famous cancer related-pathways (GSCALite), and the drug resistance analysis of common genes based on GDSC drug data. **(A)** Pie chart of pathway activity, **(B)** Heatmap of pathway activity, and **(C)** Heatmap of drugs correlated with six genes NUP153, SP3, SAP130, NUP98, UBN1, and SENP1.

Pathway activity results showed SENP1, SAP130, NUP98, and NUP153 are associated with activation of cell cycle, and SAP130 & UBN1 are associated with activation of RTK signaling. NUP153 is also associated with the strong activation of apoptosis, and SAP130 is associated with the strongest activation of DNA Damage Response, Hormone AR, and RTK signaling (Figures 7A,B).

After receiving a list of drugs related to the SENP1 from the PharmacoDB database, we obtained target and the target pathways of drugs with *p* < 0.01. After the initial investigation, we deleted drugs without target and target pathway, selected standard coefficient >0.1 and < −0.1, and then we displayed drugs as a heatmap in Figure 7C. Among them, SENP1 was sensitive to thirty-five drugs and was resistant to twenty drugs.

Among these drugs, SENP1 is sensitive to apoptosis regulators: Venetoclax, Navitoclax, pac-1, Obatoclax Mesylate, TW 37, XMD 13–2, and YM-155. Venetoclax (Tse et al., 2008) through Bcl2, Navitoclax through Bcl2, Bcl-XL, and Bcl-W, Obatoclax Mesylate through Bcl2, Bcl-XL, Bcl-W, and Mcl-1, pac-1 through Procaspase-3 and Procaspase-7, TW 37 through Bcl2, Bcl-XL, and Mcl-1, XMD13-2 through RIPK1, and YM-155 through BIRC5 (Saleem et al., 2013) regulate apoptosis. Then SENP1 may regulate apoptosis.

Mutations of RTK-signaling often cause cell transformation which was observed in a widevariety of cancers (Regad, 2015). SENP1 is sensitive to regulators of RTK-signaling pathway: Axitinib, AZD4547, BMS-754807, Crizotinib, LFM-A13, and Sorafenib. Axitinib with targeting PDGFR, KIT, and VEGFR, AZD4547 with targeting FGRF1, FGFR2, and FGFR3, BMS-754807 with targeting IGF1R and IR, Crizotinib with targeting MET, ALK, and ROS1, LFM-A13 with targeting VEGFR1, VEGFR2, VEGFR3, CSF1R, FLT3, and KIT, OSI-930 with targeting KIT, and Sorafenib with targeting PDGFR, KIT, VEGFR, and RAF regulate RTK-signaling pathway. Then SENP1 may regulate the RTK-signaling pathway.

SENP1 is sensitive to cell cycle regulators, including Bi-2536, CAY10618, GW843682X, NSC-207895, and Rigosertib. Bi-2536 with effect on PLK1-3, CAY10618 with effect on PPM1D, GW843682X with effect on PLK1, NSC-207895 with effect on PLK3, and Rigosertib with effect on CDK2, CDK7, and CDK9 regulate the cell cycle. Then we suggested SENP1 also regulates the cell cycle. These findings distinguish that SENP1 is associated with alterations of multiple oncogenic pathways.

*SENP*1 overexpression also led to resistance to drugs Paclitaxel, GSK1120212, PD-0325901 (5Z)-7-Oxozeaenol, Selumetinib, Tanespimycin, AZ628, Saracatinib, Afatinib, Gefitinib, BMS-536924, Sorafenib, KIN001-055, Bexarotene, LFM-A13, Midostaurin, Pluripotin, VNLG/124, and Dasatinib.

#### Virtual Screening of Sentrin Specific-Protease 1 With FDA-Approved Drugs

We carried out SBVS using the docking method on FDA-Approved drugs of the ZINC15 database to recognize new SENP1 inhibitors. Molecular docking is the best method to quickly estimate the binding conformations of ligands that are energy-efficient to interact with a pharmacological receptor site and has obtained popularity as a tool to store time and costs in the pipeline of drug discovery and development (Kaushik et al., 2020; Caliskan et al., 2021). ZINC15 joins biological activities of drugs, gene products, and natural products with commercial availability (Irwin and Shoichet, 2005). We supposed that, if we manage to break through the interactions that are activated, we might model a strategy to cure the disease. For this purpose, we considered SENP1 with activated interactions in the tumor state as potential drug targets. After the molecular docking was complete, the top twenty ranked results were docked using autodock4 (Table 1).

**TABLE 1:** PyRx binding energy and AutoDock binding energy of twenty top FDA-Approved results of SBVS between FDA-Approved drugs of ZINC15 and SENP1. The bold values are five compounds azilsartan, telmisartan, nilutinib, paliperidone and risperdal.

**Number**	**Compound ID**	**PyRx binding energy**	**AutoDock binding energy**
1	**ZINC000006716957**	−7.7	−6.45
2	**ZINC000014210642**	−7.4	−6.87
3	**ZINC000004214700**	−7.3	−6.82
4	ZINC000001481956	−7.2	−5.00
5	ZINC000027428713	−7.2	−5.39
6	**ZINC000001530886**	−7.1	−7.69
7	ZINC000003831490	−7.1	−6.29
8	ZINC000003932831	−7.1	−6.35
9	ZINC000009212428	−7.1	−6.30
10	ZINC000035328014	−7.1	−4.46
11	**ZINC000000538312**	−7.1	−7.21
12	ZINC000002005305	−7	−5.52
13	ZINC000003817234	−7	−4.78
14	ZINC000003913937	−7	−5.88
15	ZINC000011681563	−7	−5.05
16	ZINC000028957444	−7	−5.49
17	ZINC000052716421	−7	−5.37
18	ZINC000001540998	−6.9	−5.25
19	ZINC000003797541	−6.9	−6.40
20	ZINC000003810860	−6.9	−5.05

#### Molecular Docking *via* AutoDock Tools

After SBVS, the compounds ranked with the lowest binding energy. Twenty top results of SBVS were used for molecular docking using AutoDock4 (Table 1). Then, among the top Twenty compounds, the compounds with the lowest SBVS binding energy and the lowest AutoDock binding energy were included Nilotinib (ZINC000006716957), Azilsartan medoxomil (ZINC000014210642), Paliperidone (ZINC000004214700), Telmisartan (ZINC000001530886), and Risperdal (ZINC000000538312). Nilotinib is chronic myeloid leukemia (CML) tyrosine kinase inhibitor that was also introduced as an inhibitor of COVID-19 (Rahman et al., 2020; Singh et al., 2020). Paliperidone that can inhibit COVID-19 (Gul et al., 2020), was also demonstrated can use to Huntington treatment. Telmisartan can also be used in COVID-19 treatment (Barage et al., 2020), and Risperidal can be used for MALT1-driven cancer or autoimmune diseases (Zhang et al., 2019).

**FIGURE 8:**
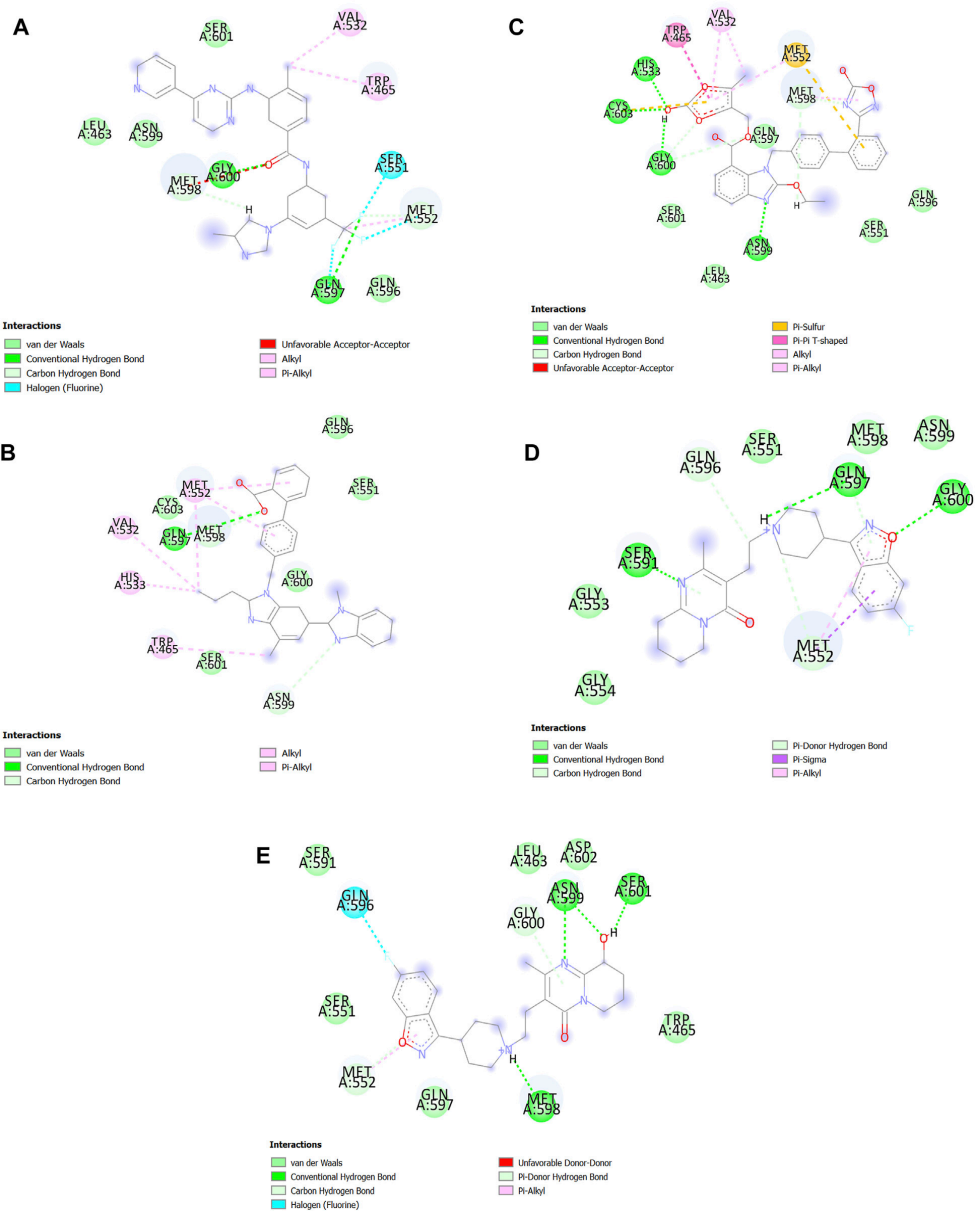
2D structure of the best drugs for SENP1 inhibition. **(A)** Nilotinib, **(B)** Telmisartan, **(C)** Azilsartan medoxomil, **(D)** Risperdal, and **(E)** Paliperidone.

In the end, the best pose of the five best AutoDock results was imported into Discovery Studio (Studio, 2008) to be visualized as a 2D structure (Figure 8). We observed which Azilsartan medoxomil constitutes the most bonds especially hydrogen bonds with the active site of SENP1. Telmisartan also constitutes the most bonds with the active site of SENP1 especially van der Waals bonds (Figure 8).

## Discussion

Cancer is the second cause of death universally. Cancer has been led to approximately 9,958 133 million deaths in 2020 according to statistics by the Global Cancer Observatory. Numerous important advances in cancer research have manifested in the genetics and pathologies of malignant tumors, which, in turn, assist the development of new anticancer agents (Dong et al., 2019). SENP1 is located on the chromosomal position 12q13.11 (Kim and Baek, 2009). Activation of transcription factors by SUMOylation and their inactivation by deSUMOylation are performed (Chang et al., 2012). DeSUMOylation of HIF1-α by SENP1 under conditions of hypoxia is needed for the stabilization of HIF1-α and the expression of HIF1-α target genes. In the mitotic cells, the knockdown of *SENP1* delays sister chromatid separation at metaphase (Nayak and Müller, 2014). The findings indicated RNA interference via *SENP1* repression leads to a global increase in SUMOylated proteins and in the number of nuclear PML bodies plus P53-mediated transcription activity that results in premature senescence (Andreou and Tavernarakis, 2010). Overexpression of SENP1 was reported in many cancers (Brems-Eskildsen et al., 2010; Xu et al., 2011; Wang et al., 2013a; Wang et al., 2013b; Ma et al., 2014; Wang et al., 2016; Zhang et al., 2016).

The study of expression, functions and molecular mechanisms of SENP1 in carcinogenesis for prognosis and treatment in cancers with abnormal *SENP1* expression is significant. Our study indicates the use of computational biology methods to explore and clarify new molecular biology mechanisms of SENP1 in tumorigenesis. In this study, we provided evidence of gene expression, survival status, immune infiltration, transcription factors and miRNAs, pharmacogenomics, and relevant cellular pathway for SENP1 as a biomarker in cancer across the Pan-Cancer cohort.

In this study, genetic alterations were not important in the investigation of SENP1 carcinogenesis mechanism in the Pan-Cancer cohort. Immune control maintains potentially metastatic or invading cancer cells and supplies new prognostic markers and novel therapeutic targets (Fridman et al., 2010). Inducing lymphocytic infiltration in the primitive tumor usually joins with a preferred clinical outcome in patients with cancer (Pagès et al., 2010). Increasing evidences showed immune cell infiltration plays a key role in cancer progression and metastasis and could affect the prognosis of cancer patients (Bremnes et al., 2016; Zeng et al., 2020). SENP1 through control of the SUMOylation status of STAT5 plays a role in lymphocytes of B and T development (Van Nguyen et al., 2012). Macrophage activation led to a severe decrease in the amount of SUMOylated IRF8 and promotion of SENP1 in activated macrophages that trigger innate immune responses (Chang et al., 2012). CD4^+^ T cells have a key role in making the immune response to cancer. CD8^+^ T cells are also cytotoxic T lymphocytes that identify specific tumor-associated antigens on MHC class I molecules on the cancer cell and can destroy cancer cells straightly (Hiraoka et al., 2006). We observed *SENP1* has higher expression and higher immune infiltration in PAAD, ESCA, and THYM. CD4^+^ T cells, CD8^+^ T cells, and macrophages were more key-related immune cells. These are our novel findings. Then we suggest that SENP1 could affect cancer prognosis by increasing immune infiltration.

Results of the Pathological Stage Plot displayed *SENP1* plays a strong role in the pathological stages in ACC, KICH, LIHC, and OV. Whereas the results of survival showed high expression of SENP1 was associated to poor prognosis of OS in the ACC, KIRP, LIHC, and THCA, and DFS in the ACC, KICH, LIHC, and MESO. Such cancer type-dependent differences in the regulation of the SENP1 could be important to the development of therapies that target SENP1.

Our study also found TFs with the highest positive correlation in UVM, and THYM, whereas miRNAs with the most inhibitory effect in KIRP, KICH, and DLBC. These TFs were involved in cell cycle and apoptosis regulation, senescence, and carcinogenesis. For example, KDM5A causes cancer through interference in the cell cycle and senescence via regulation of p16 and p27 and prevents p^RB^ function and P53 signaling in the cell cycle (Shokri et al., 2018). YY1 is an important regulator in tumorigenesis that its expression was disturbed in many tumors (Arribas et al., 2015). Since, SENP1 is regulated by these TFs, then these can be representative a carcinogenic role of the SENP1 in cancer.

With the pathway activity study, we observed SENP1 activates cell cycle. Consistent with the pathway activity, enrichment analysis indicated SENP1-correlated genes and SENP1-binding genes were primarily complicated in the cell cycle. This was more verified by the pharmacogenomic data from pharmacoDB and GDSC that SENP1 could widely affect anti-cancer drug sensitivity across TCGA cancer types. Because SENP1 is sensitive to drugs complicated in the cell cycle. Cell cycle disorder participates in aberrant proliferation, decreased apoptosis, invasion, and metastasis (Zaretsky et al., 2016). Then, SENP1 with intervening in the cell cycle cause cancer. These findings may be protection for drug-targeted therapy in cancer.

Pathway activity also showed SENP1-correlated and SENP1-binding genes: SAP130, NUP98, and NUP153 are associated with activation of the cell cycle, UBN1 is associated with the strongest activation of RTK signaling, NUP153 is associated with the strongest activation of apoptosis, and SAP130 is associated the strongest activation of DNA Damage Response, and Hormone AR. SENP1 is also sensitive to the apoptosis and RTK signaling regulators. As well as, SENP1-correlated and SENP1-binding genes facilitate tumorigenesis by interfering in apoptosis, and DNA Damage stimulus. Then SENP1, and SENP1-associated genes increase carcinogenesis via different mechanisms.

We first reported increased *SENP1* expression in ESCA, DLBC, THYM, and CHOL. We also shown *SENP1* overexpression cause resistance to drugs Paclitaxel, GSK1120212 (Trametinib), PD-0325901, (5Z)-7-Oxozeaenol, Selumetinib, Tanespimycin, AZ628, Saracatinib, Afatinib, Gefitinib, BMS-536924, Sorafenib, KIN001-055, Bexarotene, LFM-A13, Midostaurin, Pluripotin, VNLG/124, and Dasatinib. The studies shown among these drugs, Paclitaxel can apply to treat B-cell lymphoma (Nevala et al., 2017), GBM (Zhan et al., 2010), PAAD (Ma and Hidalgo, 2013), ESCA (Gong et al., 2009), CHOL (Hirose et al., 2013; Cadamuro et al., 2016; Sahai et al., 2018), and THYM (Umemura et al., 2002). Other drugs also include GSK1120212 to treat CHOL (Loaiza-Bonilla et al., 2014), and PAAD (Walters et al., 2013; Estrada-Bernal et al., 2015), PD-0325901 to treat PAAD (van Geel et al., 2020), (5Z)-7-Oxozeaenol to treat DLBC (Bhalla et al., 2011), Selumetinib to treat CHOL (Prado et al., 2012), Afatinib to treat GBM (Alshami et al., 2015), CHOL (Zhang et al., 2018), PAAD (Ioannou et al., 2011; Ioannou et al., 2013), and ESCA (Wong et al., 2015), Gefitinib to treat PAAD (Li et al., 2004), GBM (Aljohani et al., 2015; Mu et al., 2016), CHOL (Yang et al., 2015), and ESCA (Guo et al., 2006), BMS-536924 to treat GBM (Zhou, 2015), and ESCA (Adachi et al., 2014), Sorafenib to treat CHOL (Huether et al., 2007), GBM (Jo et al., 2018), ESCA (Delgado et al., 2008), PAAD (Siu et al., 2006; Rausch et al., 2010), and DLBC (Greenwald et al., 2013), Bexarotene to treat GBM (Heo et al., 2016), and Dasatinib to treat DLBC (Cann et al., 2019; Scuoppo et al., 2019), THYM (Chuah et al., 2006), PAAD (Chang et al., 2008), and ESCA (Chen et al., 2015). Then SENP1 inhibition while using these drugs can be a suitable therapy strategy.

On the other hand, among the FDA-approved drugs we found inhibitors for the SENP1 including Nilotinib, Telmisartan, Azilsartan medoxomil, Risperdal, and Paliperidone. The studies indicated Telmisartan can be used to treat ESCA (Matsui et al., 2019), EAC (Fujihara et al., 2017), CHOL (Samukawa et al., 2017), and hematologic malignancies (Kozako et al., 2016). It has also been reported that Telmisartan can apply in the treatment of endometrial (Koyama et al., 2014), lung cancers (Rasheduzzaman et al., 2018), bladder and urological (Matsuyama et al., 2010), ovarian (Pu et al., 2016), colon (Lee et al., 2014), renal (de Araújo Júnior et al., 2015), prostate (Funao et al., 2008), gastric (Fujita et al., 2020), and breast (Kociecka et al., 2010) cancers, hepatocellular carcinoma (Oura et al., 2017), and GBM (Wang et al., 2021). Nilotinib can also be used to treat CHOL (Marin et al., 2018), DLBC (Robak and Robak, 2012; Cai et al., 2019), and THYM (Kelly, 2013; Simonelli et al., 2015). Moreover, Nilotinib is also used to treat other cancers including ovarian (Weigel et al., 2014), gastric (Onoyama et al., 2013), liver (Frolov, 2017) cancers, ALL, AML, and CML (Bleeker and Bardelli, 2007), GBM (Au et al., 2015; Frolov et al., 2016), melanoma (Guo et al., 2017), and gastrointestinal stromal tumors (Blay et al., 2015). Risperdal is also being used to treat DLBC (Gallagher et al., 2008), and GBM (Lee et al., 2001). Paliperidone and Azilsartan also were used to GBM (Kast, 2010), and hepatocellular carcinoma (Ahmadian et al., 2018), respectively.

Therefore, these FDA-approved drugs can be used alone to treat cancer, which shows they can be applied to treat cancer and to inhibit SENP1. So, these inhibitors can use with drugs that cause drug resistance of SENP1.

This study presents evidence of the associations between the expression of SENP1 and cancer immunity. Consistent with this finding, we have seen that SENP1 correlates with immune infiltration and several TFs increase the SENP1 expression in cancer. We also exhibited that SENP1 is highly correlated with sensitivity and resistance to anti-cancer drugs and drug-targeted genes across cancer cell lines. Our results provide a new document about the role of SENP1 in tumorigenesis and new insights into cancer therapy targets. The functions of SENP1 and associated genes were primarily complicated in the tumor-related functions and pathways that show SENP1 may mediate the progression and tumorigenesis of cancer. The FDA-Approved drugs concomitant to chemotherapy drugs do better treatment.

These findings propose the clinical value of evaluating SENP1 for specific cancer diagnosis and treatment decisions. Experimental work is necessary to further analyze and validate these findings.

## Data Availability

The raw data supporting the conclusion of this article will be made available by the authors, without undue reservation.
