# Supplementary material for: Evidence of Omics, Immune Infiltration, and Pharmacogenomic for SENP1 in the Pan-Cancer Cohort
Source: Front Pharmacol. 2021 Jul 1;12:700454. doi: 10.3389/fphar.2021.700454 (PMC8280523; doi:10.3389/fphar.2021.700454)
Supplement: Supplementary file 1 [file DataSheet1.docx]

Supplementary Material

Evidence of Omics, Immune Infiltration, and Pharmacogenomic for SENP1 in the Pan-Cancer Cohort

## *
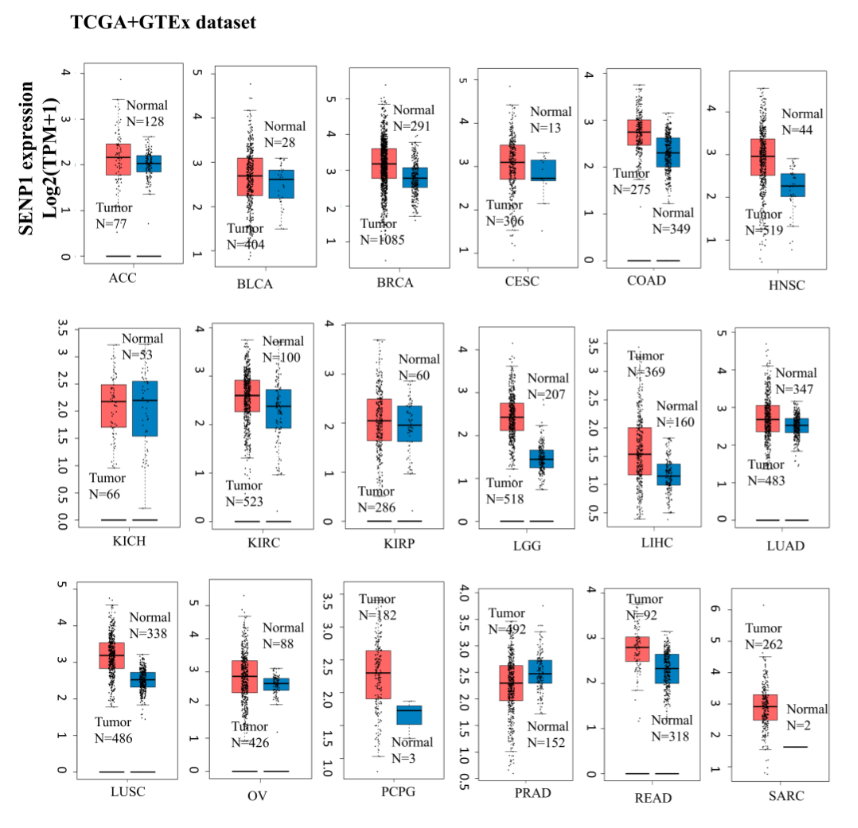
Supplementary Figures*


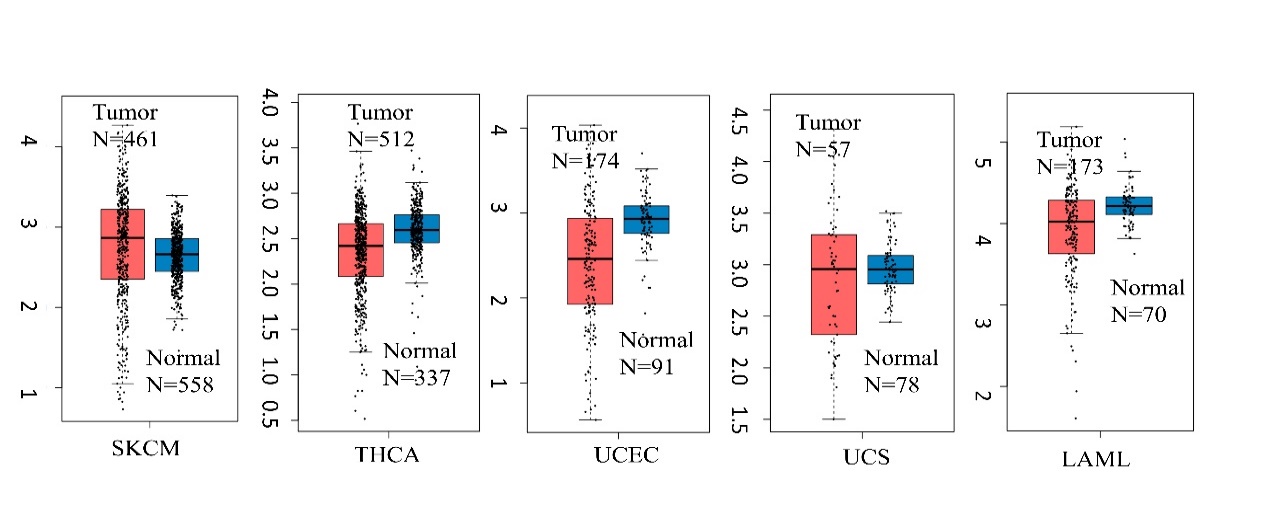


**Supplementary Figure 1:** Box plots of *SENP1* expression in tumor tissues compared with normal tissues in cancers: BLCA, BRCA, CESC, COAD, HNSC, KICH, KIRC, KIRP, LGG, LIHC, LUAD, LUSC, OV, PCPG, PRAD, READ, SARC, SKCM, THCA, UCEC, UCS, and LAML with *P*>0.01


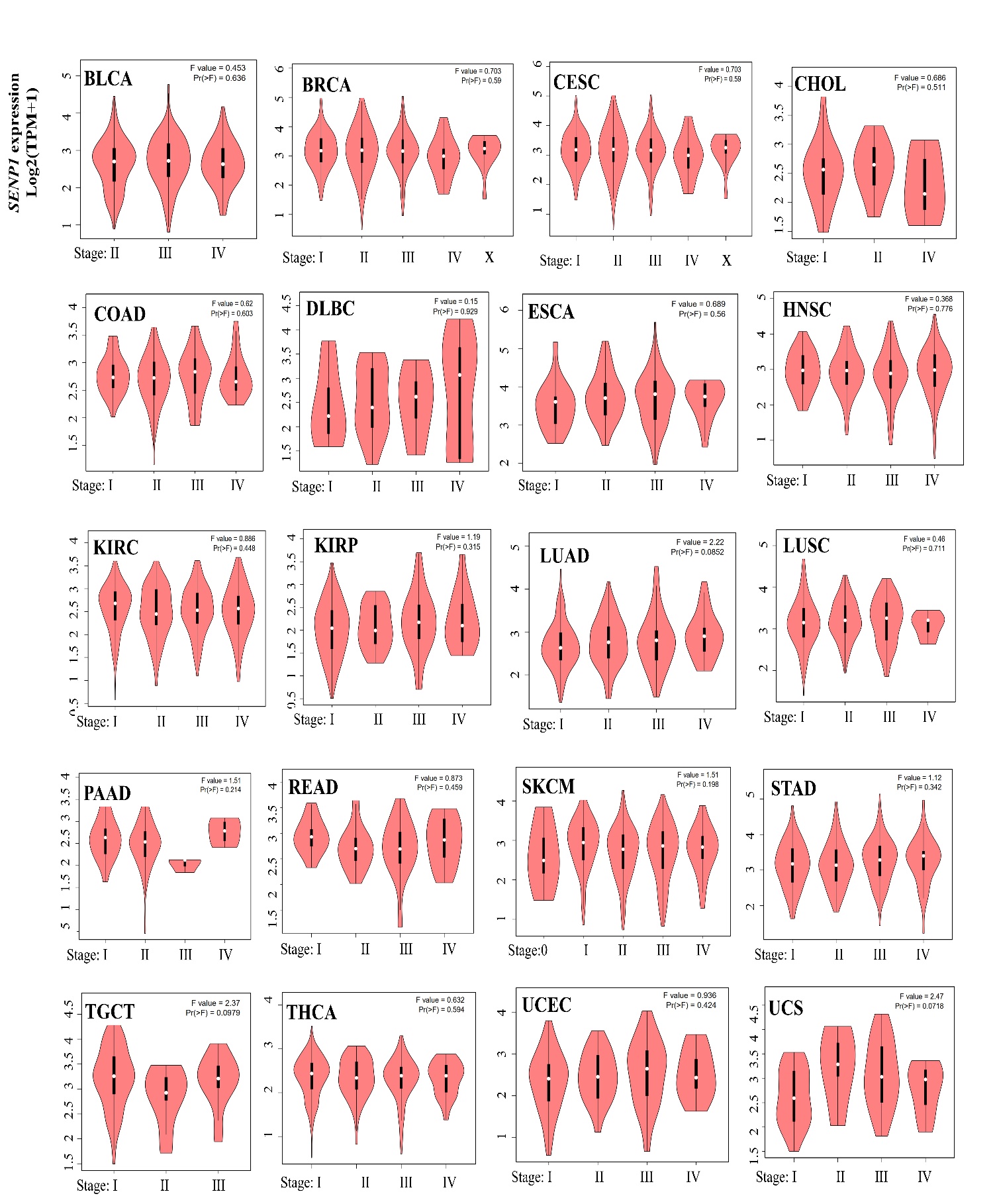


**Supplementary Figure 2:** *SENP1* expression in different stages in tumor tissues compared with normal tissues in cancers: BLCA, BRCA, CESC, CHOL, COAD, DLBC, ESCA, HNSC, KIRC, KIRP, LUAD, LUSC, PAAD, READ, SKCM, STAD, TGCT, THCA, UCEC, and UCS with *P*>0.05


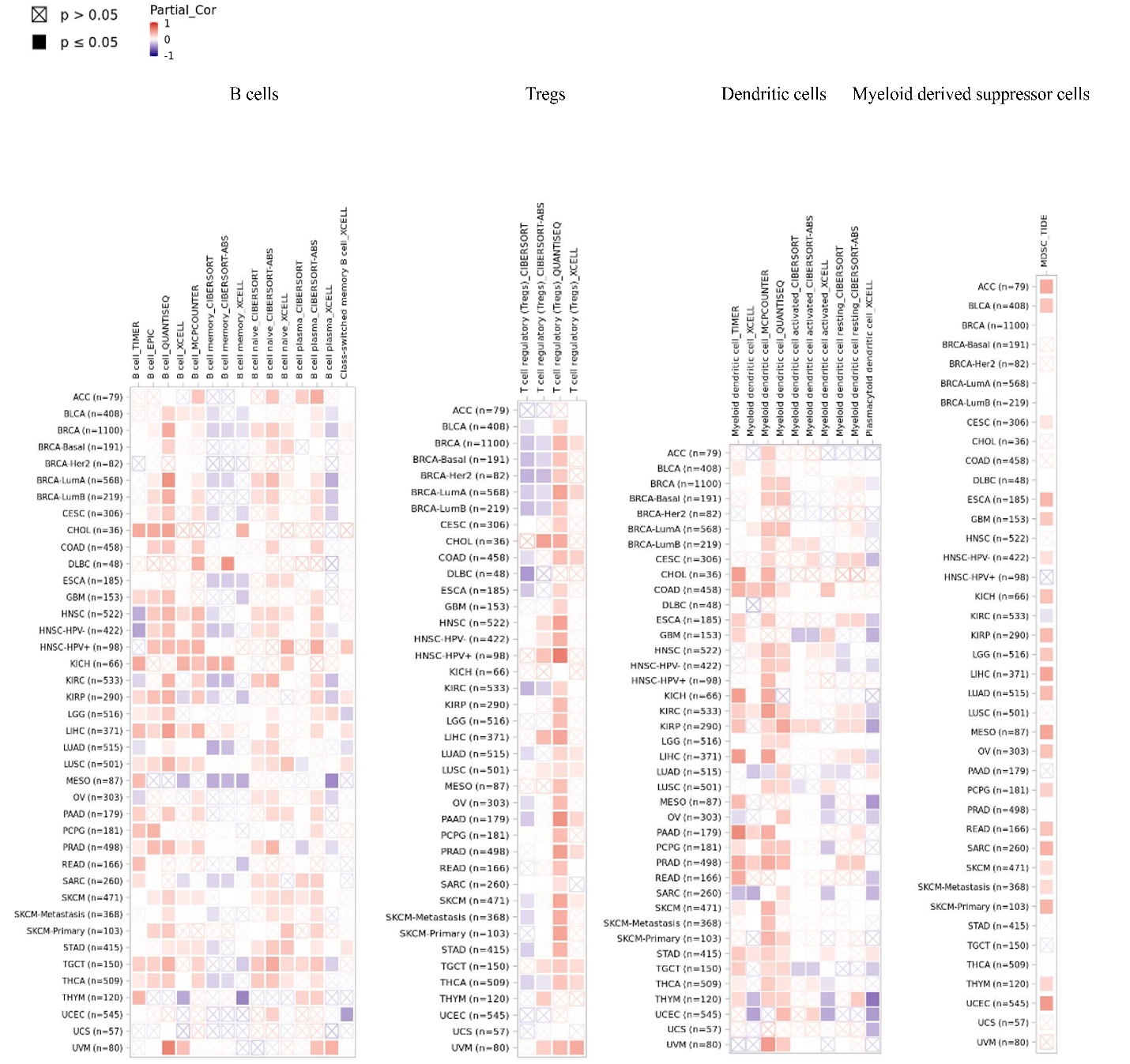


**Supplementary Figure 3**: Heatmap of immune cells (B cells, Tregs, dendritic cells, and myeloid derived suppressor cells) with positive significant correlation with SENP1 in Pan-Cancer


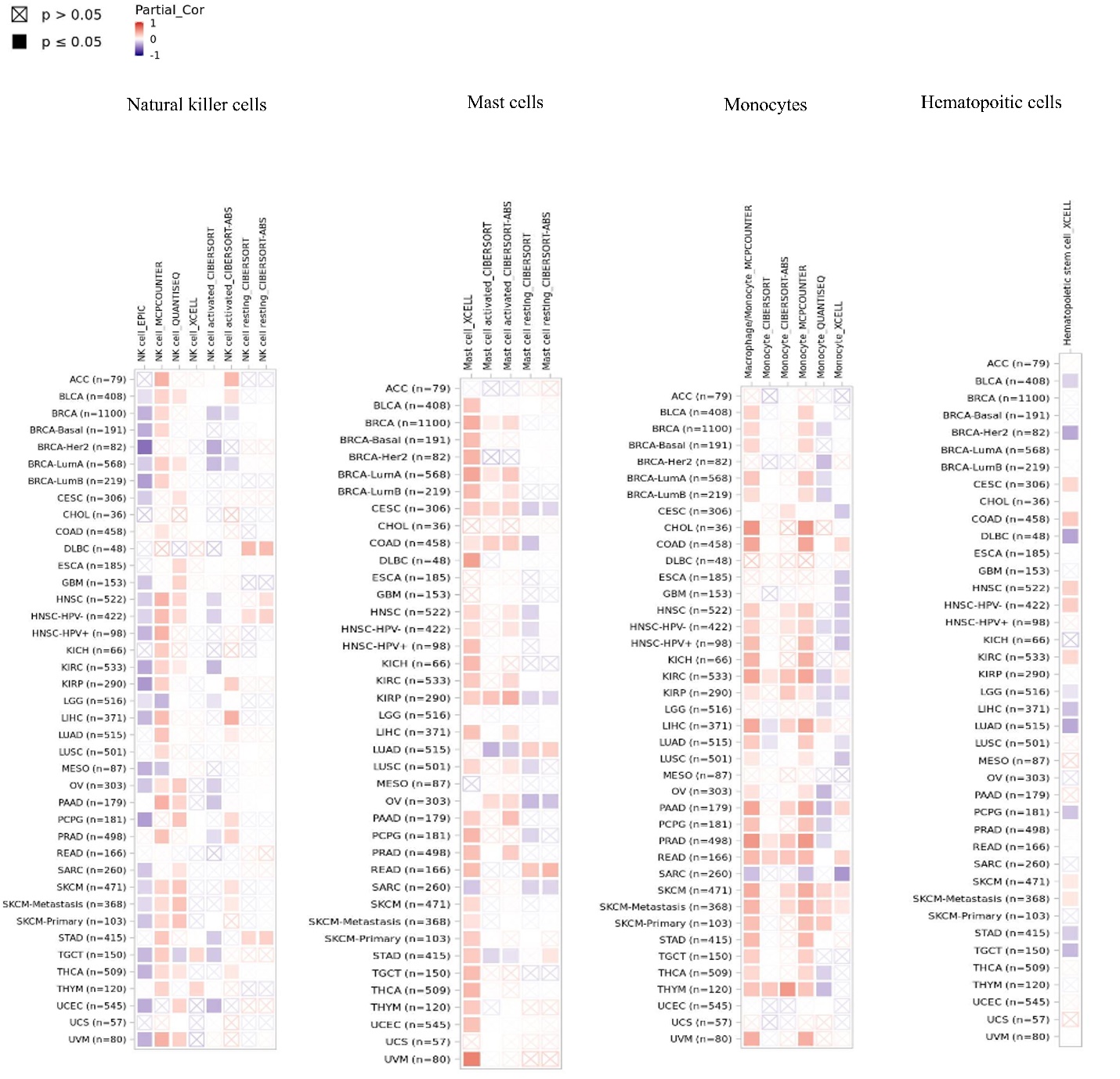


**Supplementary Figure 3**: Heatmap of immune cells (Natural killer cells, mast cells, monocytes, and hematopoietic cells) with positive significant correlation with SENP1 in Pan-Cancer


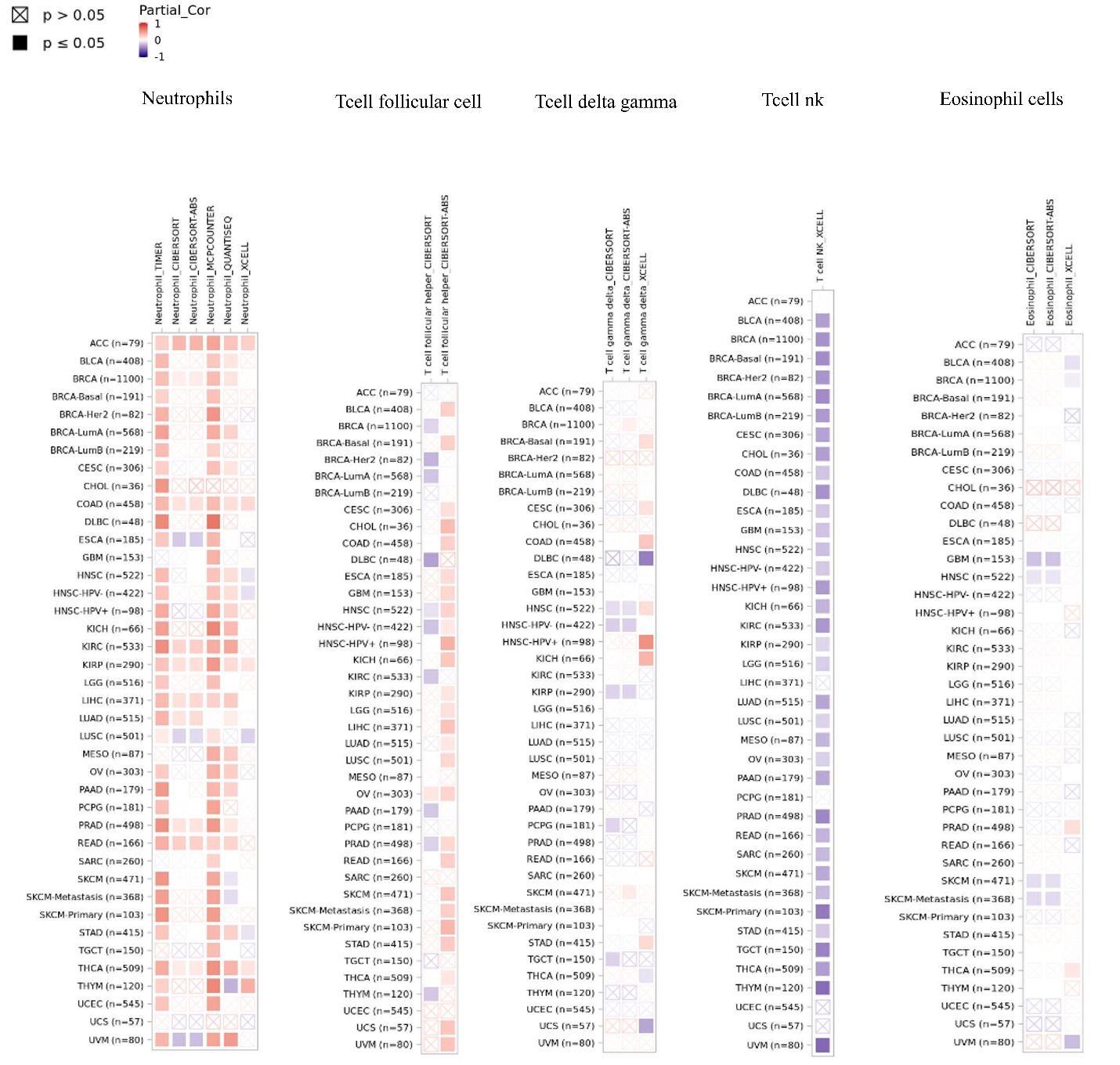


**Supplementary Figure 3**: Heatmap of immune cells (neutrophils, Tcell follicular cells, Tcell delta gamma, Tcell NK, and eosinophils) with positive significant correlation with SENP1 in Pan-Cancer


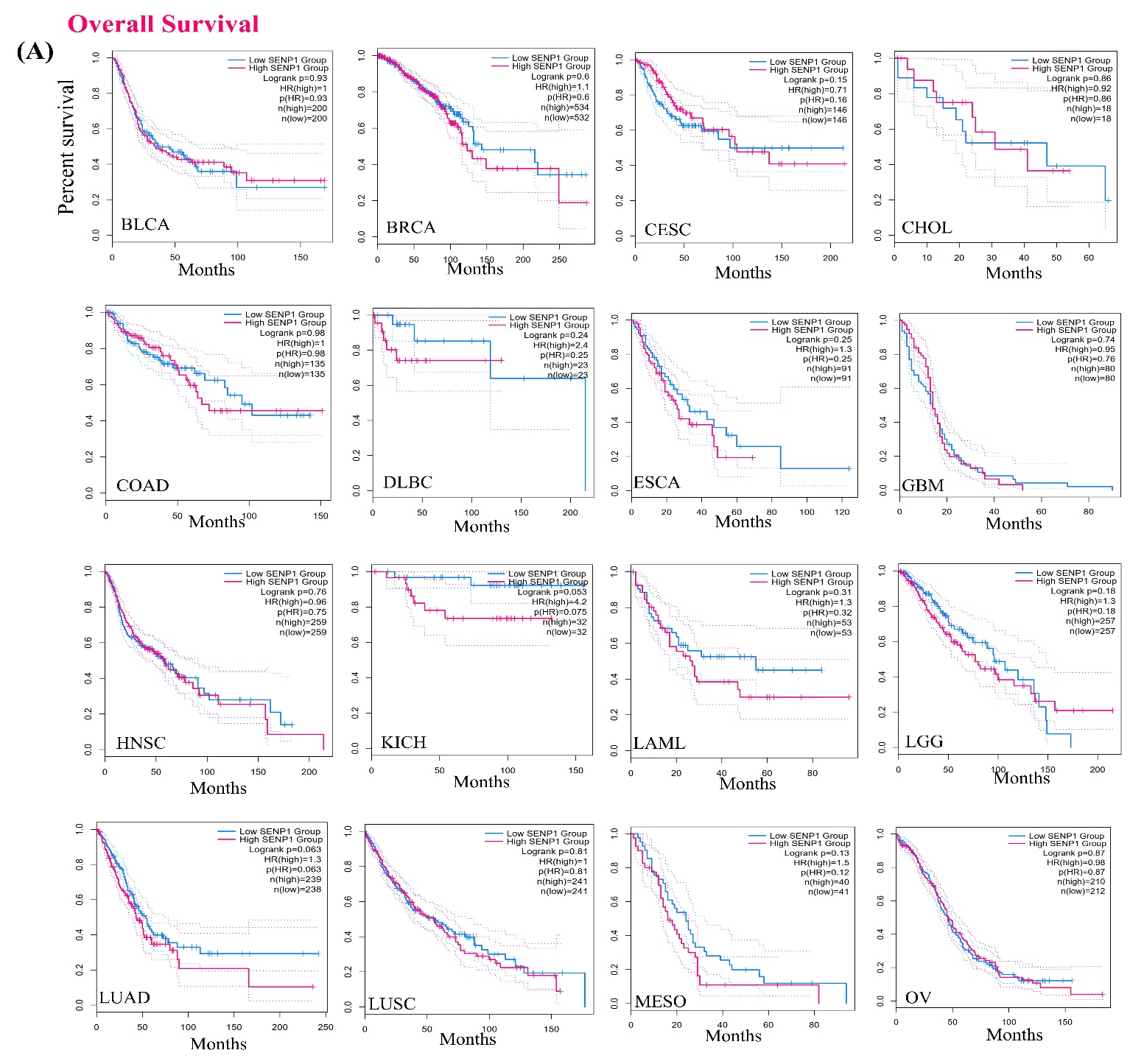


**Supplementary Figure 4:** Survival of patients with *SENP1* changed expression in Pan-Cancer. a) Overall Survival of tumor tissues with *SENP1* expression compared with normal tissues in BLCA, BRCA, CESC, CHOL, COAD, DLBC, ESCA, GBM, HNSC, KICH, LAML, LGG, LUAD, LUSC, MESO, and OV with *P*>0.05


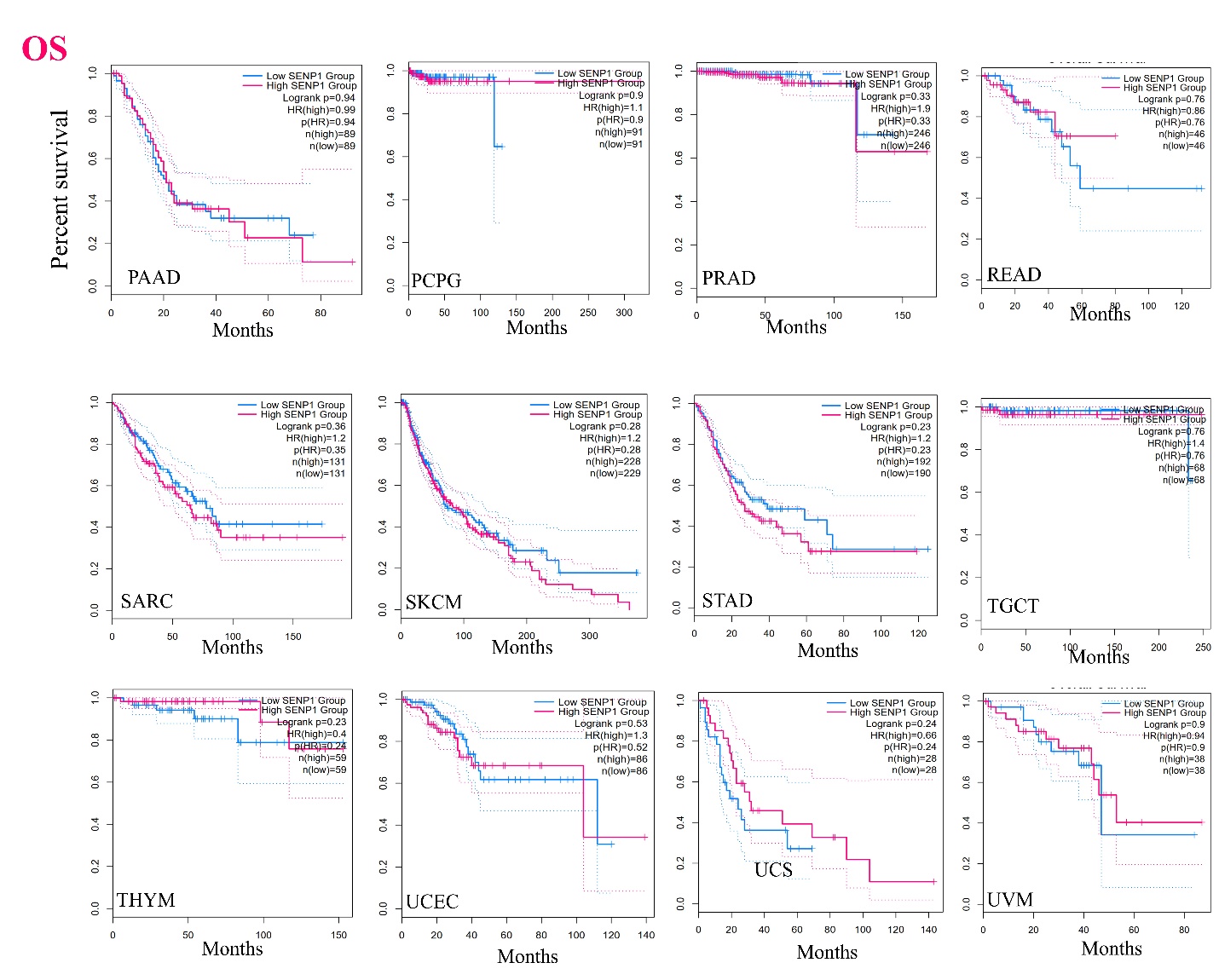


**Supplementary Figure 4:** Survival of tumor tissues with *SENP1* changed expression in Pan-Cancer. a) Overall Survival of tumor tissues with *SENP1* expression compared with normal tissues in PAAD, PCPG, PRAD, READ, SARC, SKCM, STAD, TGTC, THYM, UCEC, UCS, and UVM with *P*>0.05


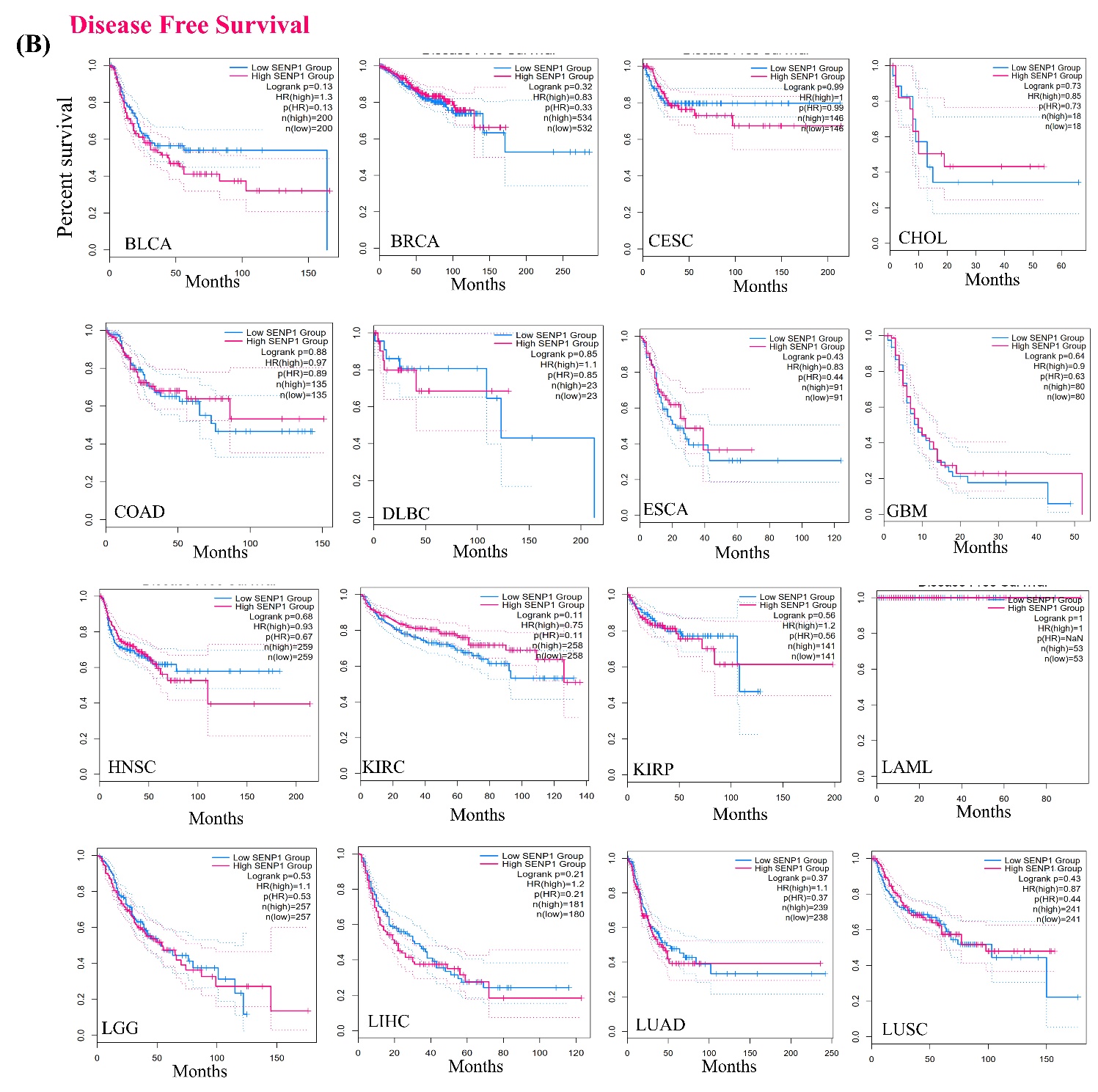


**Supplementary Figure 4:** Disease Free Survival of tumor tissues with *SENP1* expression compared with normal tissues in BLCA, BRCA, CESC, CHOL, COAD, DLBC, ESCA, GBM, HNSC, KIRC, KIRP, LAML, LGG, LIHC, LUAD, and LUSC with *P*>0.05


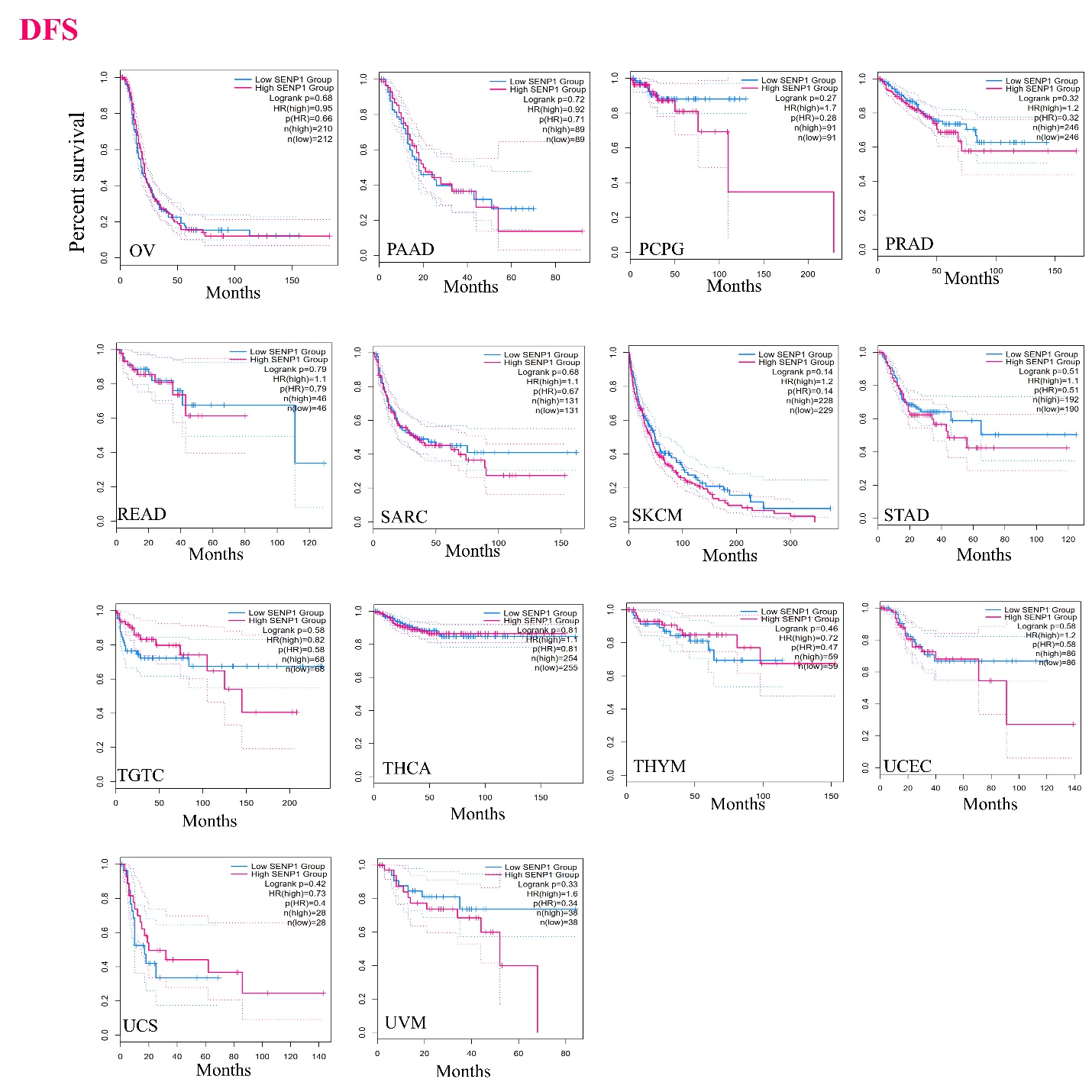


**Supplementary Figure 4:** Disease Free Survival of tumor tissues with *SENP1* expression compared with normal tissues in OV, PAAD, PCPG, PRAD, READ, SARC, SKCM, STAD, TGTC, THCA, THYM, UCEC, UCS, and UVM with *P*>0.05


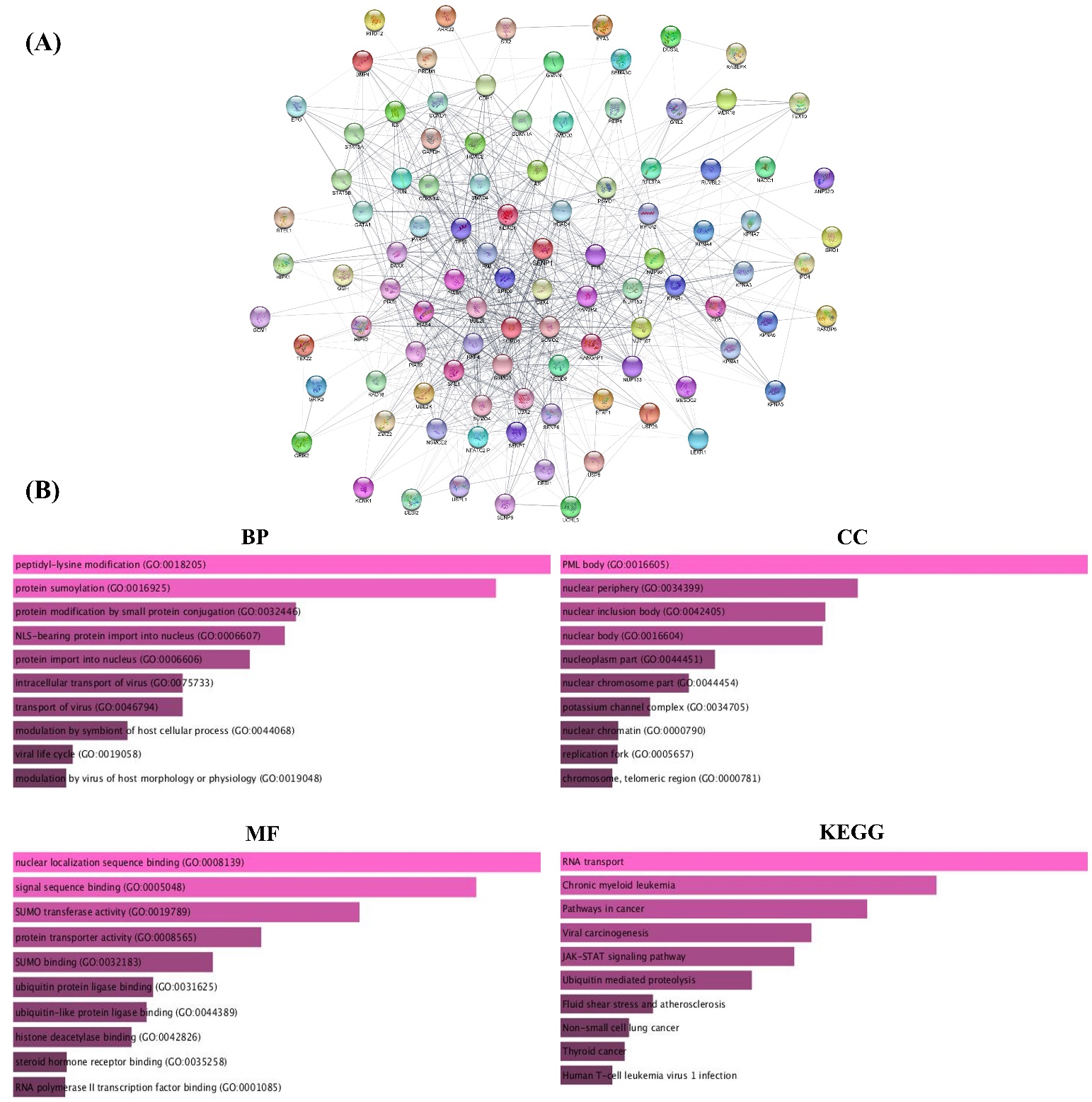


**Supplementary Figure 5:** SENP1-binding genes and enrichment. A) STRING network, B) Go ontology and KEGG pathway enrichment


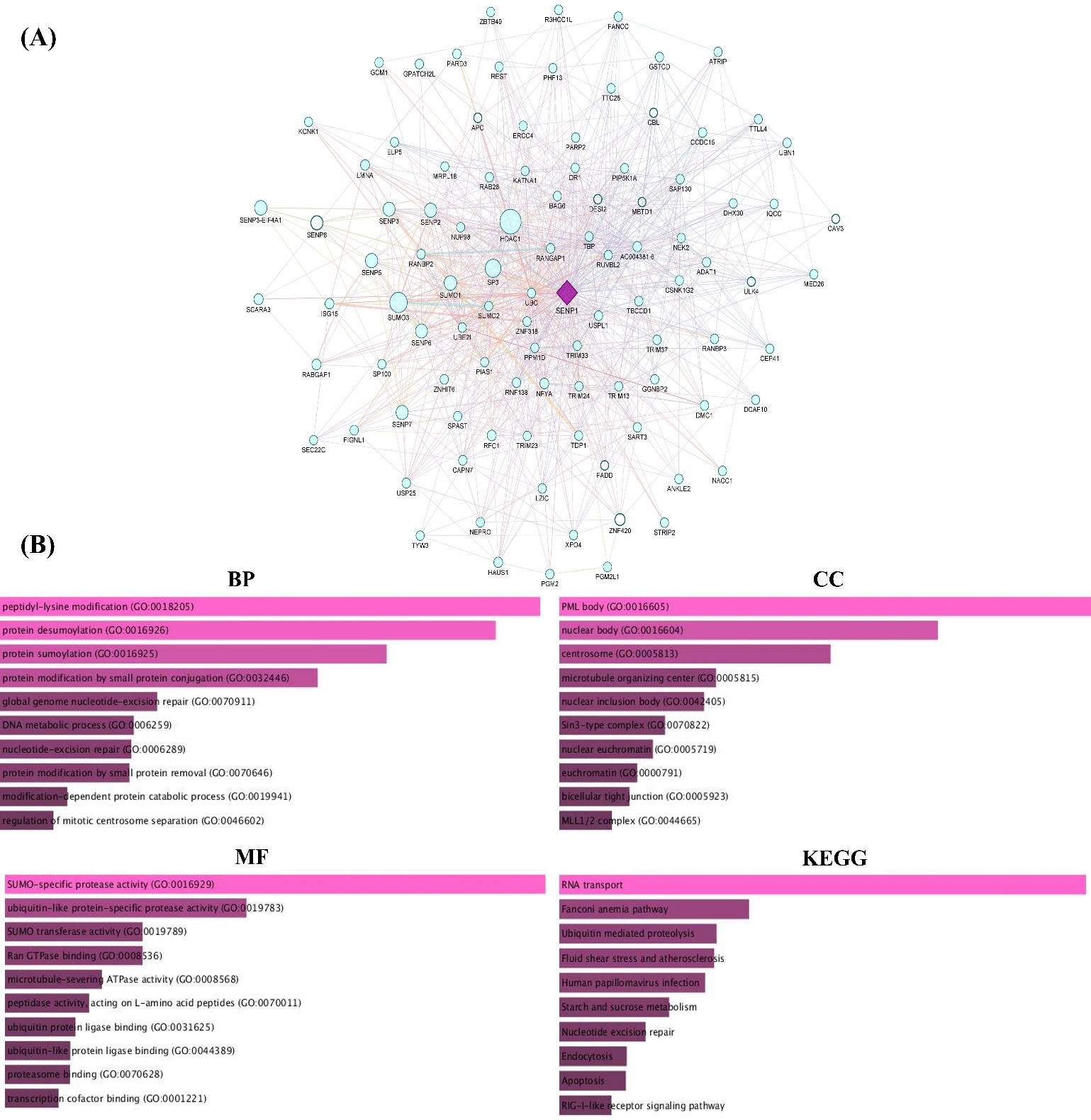


**Supplementary Figure 6:** SENP1-binding genes network and enrichment. A) GeneMANIA network, B) Go ontology and KEGG pathway enrichment

**Supplementary Figure 6:** SENP1-binding genes network and enrichment. A) GeneMANIA network, B) Go ontology and KEGG pathway enrichment
